# Intermittent fasting reduces neuroinflammation in intracerebral hemorrhage through the Sirt3/Nrf2/HO-1 pathway

**DOI:** 10.1186/s12974-022-02474-2

**Published:** 2022-05-27

**Authors:** Shuhui Dai, Jialiang Wei, Hongchen Zhang, Peng Luo, Yuefan Yang, Xiaofan Jiang, Zhou Fei, Wenbin Liang, Jianli Jiang, Xia Li

**Affiliations:** 1 Department of Neurosurgery, Xijing Hospital, Fourth Military Medical University, 127 Changlexi Road, Xi’an, China; 2 Department of Health Service, Fourth Military Medical University, Xi’an, China; 3 University of Ottawa Heart Institute, Department of Cellular and Molecular Medicine, University of Ottawa, Ottawa, ON Canada; 4 National Translational Science Center for Molecular Medicine and Department of Cell Biology, Fourth Military Medical University, 169 Changlexi Road, Xi’an, China

**Keywords:** Intracerebral hemorrhage, Intermittent fasting, Sirt3, Microglia, Inflammation

## Abstract

**Background:**

Inflammation contributes to the poor prognosis of intracerebral hemorrhage (ICH). Intermittent fasting (IF) has been shown to be protective against inflammation in multiple pathogenic processes. In the present study, we aimed to investigated the beneficial effects of IF in attenuating neuroinflammation and neurological deficits in a mouse model of ICH and to investigate the underlying mechanism.

**Methods:**

ICH was modeled by intrastriatal injection of autologous blood and IF was modeled by every-other-day feeding in male control mice (C57BL/6), mice with and microglia specific knockout Sirt3^f/f^;Cx3cr1-Cre (Sirt3 cKO), and Sirt3^f/f^ (wild-type) mice. Brain tissues and arterial blood were harvested at 1, 3, 7 and 28 days after ICH for immunohistochemistry analysis of Iba-1, DARPP-32 and HO-1, morphological analysis by HE staining and inflammatory factor release tests by ELISA. Neurological functions were approached by corner test and cylinder test. Fluorescent double-labeled staining of Iba-1 with CD16, Arg1 or Sirt3 was used to provide direct image of co-expression of these molecules in microglia. TUNEL, cleaved caspase-3 and Nissl staining was performed to evaluate cellular injuries.

**Results:**

IF alleviated neurological deficits in both acute and chronic phases after ICH. Morphologically, IF enhanced hematoma clearance, reduced brain edema in acute phase and attenuated striatum atrophy in chronic phase. In addition, IF decreased the numbers of TUNEL^+^ cells and increased Nissl^+^ neuron number at day 1, 3 and 7 after ICH. IF suppressed CD16^+^Iba-1^+^ microglia activation at day 3 after ICH and reduced inflammatory releases, such as IL-1β and TNF-α. The above effects of IF were attenuated by microglia Sirt3 deletion partly because of an inhibition of Nrf2/HO-1 signaling pathway. Interestingly, IF increased Iba-1^+^ microglia number at day 7 which mainly expressed Arg1 while decreased the proinflammatory factor levels. In mice with microglia-specific Sirt3 deletion, the effects of IF on Iba-1^+^ microglia activation and anti-inflammatory factor expressions were attenuated when compared with wild-type Sirt3^f/f^ mice.

**Conclusions:**

IF protects against ICH by suppressing the inflammatory responses via the Sirt3/Nrf2/HO-1 pathway.

**Supplementary Information:**

The online version contains supplementary material available at 10.1186/s12974-022-02474-2.

## Background

Intracerebral hemorrhage (ICH) accounts for 10–15% of all stroke cases which are characterized with 30–67% mortality and poor prognosis [1]. Neurosurgical clot evacuation is currently the primary treatment for ICH [2]. However, clinical trials did not show a clear benefit of hematoma evacuation over conservative management [3, 4]. Thus, it is needed to further investigate the mechanisms if ICH so that more efficient therapeutic strategies could be developed.

During the secondary brain injury after ICH, neuroinflammation mediated by microglial activation plays an essential role [5]. The products of red blood cell breakdown, such as hemoglobin, heme and iron, are strong inducers for microglia activation [6]. Activated microglia play multiple roles in ICH: it exhibits a protective effect by engulfing cellular debris and deleterious products, but also leads to strong inflammatory response by releasing proinflammatory cytokines, such as interleukin (IL)-1β, IL-6, inducible nitric oxide synthase (iNOS) and tumor necrosis factor (TNF)-α [7]. Recent studies have shown that the suppression of microglia-induced inflammatory response protects brain against damages caused by ICH [8]. In addition, our previous studies have demonstrated that the inhibition of microglial activation leads to neuroprotective effects in neurological functions [9].

Intermittent fasting (IF), defined as every-other-day feeding, has been shown to extend lifespan and enhance health status in mammals [10, 11]. Recent studies have also reported that IF exerts a beneficial effect in different models of central nervous system (CNS) disorders or aged brain [12, 13]. Mechanistically, IF enhances neuronal resistance against multiple types of injuries by inducing a mild adaptive stress response, therefore improving learning, memory, brain plasticity as well as reducing inflammation and neurodegeneration [14, 15]. IF also confers a protective effect against oxidative stress and inflammation by activating antistress responses, including nuclear respiratory factor (Nrf)-1, Nrf-2 and mammalian target of rapamycin (mTOR) pathways [16, 17]. Moreover, IF increases NAD + /NADH through altering cellular metabolic processes which leads to the Sirtuin proteins activation [18]. We have recently shown that, Sirt3, which is a member of the Sirtuin family and is primarily located in mitochondria in the cells, plays an essential role in regulating cellular apoptosis and autophagy after oxidative neuronal stress [19]. However, it remains unknown if IF has any beneficial effects in ICH.

In the present study, we investigated the role of IF in ICH-induced brain damage and microglial activation, as well as the involvement of Sirt3. We found that during both the acute and chronic stages of ICH, IF conferred a beneficial effect against neuronal injury through supressing the inflammation induced by microglia activation. In this process, microglial Sirt3 reduced the release of proinflammatory factors, therefore attenuating neuroinflammation response and neurological deficits through activating Nrf2/HO-1 signaling pathway. This study suggests that IF and Sirt3 modulation are potential therapeutic strategies for ICH.

## Methods

### Animals

All the mice used in this study were male and at the age of 8–10 weeks. Eighty-nine C57BL/6 mice, 62 Sirt3^f/f^ mice (Wild type) and 75 Sirt3^f/f^;Cx3cr1-Cre mice (Sirt3 cKO) (purchased from Shanghai Model Organisms, China) were randomly assigned into different experimental groups according to different study designs (details were available in Tab S9). Mice were housed in a 12-h light/dark cycle under conditions of a controlled temperature and humidity. Mice in the IF group were feed every other day with free access to water while the other groups were allowed unlimited access to food and water. All experiments were conducted according to protocols approved by the Institutional Animal Ethics Committee of Fourth Military Medical University.

### Intracerebral hemorrhage model

ICH was induced by intrastriatal injection of 30 μl autologous blood extracted from right femoral artery into the right basal ganglia as previously described [20] and details were available in the Additional file 1: Data Supplement.

### Experimental design

In experiment 1, C57BL/6 mice were randomly assigned into ICH and ICH + IF groups. Behavioral tests, Nissl staining, microglia activation measurement with Iba-1 staining and cytokine release were performed on days 1, 3, 7 and 28 after ICH. Hematoma volume, brain edema and TUNEL^+^ cells was analysed on days 1, 3 and 7. Brain atrophy was measured on day 28.

In experiment 2, C57BL/6 mice were randomly assigned into sham, ICH and ICH + IF groups. Sirt3 expression in the ipsilateral basal ganglia was measured on days 3 and 7 after ICH.

In experiment 3, Sirt3^f/f^ or Sirt3^f/f^;Cx3cr1-Cre mice were randomly assigned into ICH and ICH + IF groups. Behavioral tests were performed on days 1, 3, 7 and 28 after ICH. Hematoma volume, brain edema and HO-1/Nrf2 expression were measured on days 3 and 7. TUNEL^+^ cells was analysed on days 1, 3 and 7. Brain atrophy was measured on day 28. M1 type microglia activation was observed on day 3 while M2 type was on day 7.

In experiment 4, Sirt3^f/f^ or Sirt3^f/f^;Cx3cr1-Cre mice were randomly assigned into two groups receiving Vehicle or DMF treatment before ICH + IF for 7 days and all the data were obtained on day 3 after ICH, including hematoma volume, behavioral score and cytokine release.

Behavioral tests were performed in all animals before modeling and the images for histological analysis were obtained within 2 mm around the hematoma area in the basal ganglia (Additional file 1: Fig. S1).

### Behavioral tests

Neurological function was evaluated on days 0, 1, 3, 7 and 28 after ICH by an investigator blinded to the group assignments with corner test and cylinder test descried as previous [21, 22] and details were available in the Additional file 1: Data Supplement.

### Nissl and hematoxylin–eosin (H&E) staining

Details were available in the Additional file 1: Data Supplement.

### TUNEL staining

Cellular injury was qualified by TUNEL staining according to the manufacturer’s protocol (Roche, Switzerland). Slices were photographed with a fluorescence microscope (U-HGLGPS, OLYMPUS, Japan) under 40 × magnification. The numbers of TUNEL-positive cells were calculated with ImageJ software (version 1.8.0). Data was presented as the number of TUNEL-positive cells per square millimetre [23].

### Immunohistochemistry and immunofluorescence

Brain sections were washed with 0.01 M PBS followed by incubation with 5% BSA (Bovine Serum Albumin) blocking buffer. The sections were then incubated with primary antibodies diluted in 0.01 M PBS (seen in the Additional file 1: Data Supplement) at 4℃ overnight. Then after washed with PBS three times, slices were incubated with the secondary antibodies conjugated with IgG biotin, Alexa Fluor 488 or 594 for 2 h at room temperature. For immunofluorescence, the slides were washed 3 times and then covered with a mounting medium containing 4,6-diamidino-2-phenylindole (DAPI) (Sigma-Aldrich, F6057). A confocal microscope (FluoView FV 1000, Olympus, Japan) was used to obtain immunofluorescence microscopic images. For immunohistochemistry, sections were placed in 3,3’-Diaminobenzidine (DAB) solution containing 0.03% H_2_O_2_ for 5–8 min. For cell counting, five images per animal were taken and all the measurements were repeated three times by a blinded investigator.

### Microglia isolation

Details were available in the Additional file 1: Data Supplement.

### Enzyme-linked immunosorbent assay (ELISA)

ELISA was used to measure concentration of cytokines IL-1β (ab100704), TNF-α (ab100747), IL-10 (ab46103) and TGF-β1 (ab119557) in blood according to the manufacturer’s protocol (Abcam, USA).

### RT-PCR

Details were available in the Additional file 1: Data Supplement.

### Western blot analysis

Details were available in the Additional file 1: Data Supplement.

### Statistical analysis

All the experiments were repeated at least 3 times and data was presented as mean ± standard deviation (SD) and analysed with GraphPad Prism 8.0 (GraphPad Software, USA). The data comparison among multiple groups was performed with one-way ANOVA followed by Tukey’s post hoc test. Two-way ANOVA analysis plus post hoc Bonferroni’s test was applied for behavioral data. Student *t* test was performed to analysis data between two groups. *p* < 0.05 was considered statistically significant.

## Results

### IF attenuates hemorrhagic brain damage in mice

**Fig. 1:**
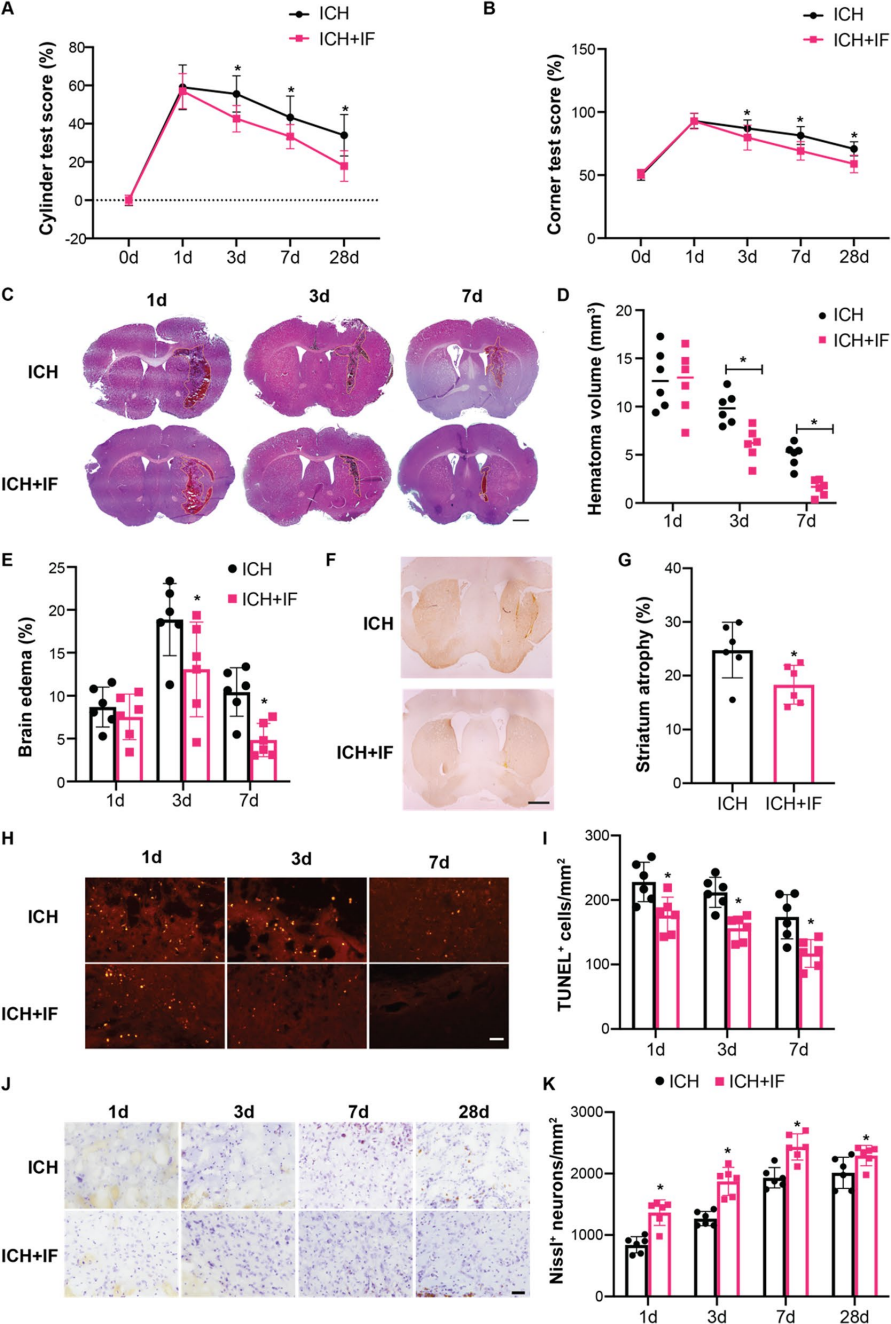
Effects of intermittent fasting on ICH-induced brain injury and neurological deficits in mice. Cylinder test **A** and corner test **B** tests were performed pre-ICH (*n* = 41–42) and at day 1 (*n* = 37–38), 3 (*n* = 27), 7 (*n* = 18) and 28 (*n* = 9) after ICH in mice with or without IF treatment. **C** Representative HE staining images of the largest clot at day 1, 3 and 7 days after ICH with or without IF treatment, Scale bar = 1 mm. Quantification of hematoma size **D** and brain volume **E** in ICH and ICH + IF groups (*n* = 6). **F** Examples of DARPP-1 immunohistochemistry in the basal ganglia at 28 day after ICH with or without IF treatment, Scale bar = 1 mm. **G** Quantification of ipsilateral shrinkage of the DARPP-32 positive area in the mice (*n* = 6). **H** TUNEL staining of ipsilateral mice brain and **I** the assessment of the number of TUNEL positive cells (*n* = 6), Scale bar = 50 μm. **J** Nissl staining of ipsilateral mice brain and **K** the assessment of the number of Nissl positive cells (*n* = 6), Scale bar = 50 μm. Statistical analysis was performed using two-way ANOVA analysis plus post hoc Bonferroni’s test for behavioural data (**A**, **B**), one-way ANOVA followed by post hoc Tukey’s test for multiple comparisons (D-K). Values are mean ± SD, *P < 0.05 compared with ICH group

To determine the effects of IF on ICH-induced brain damage, mice were assigned randomly to ICH and ICH + IF groups and sacrificed at 1, 3, 7 and 28 days after ICH onset. When compared with ICH groups, the neurological function of mice was improved significantly on an IF diet except for the day 1 (Additional file 1: Tables S1, S2, Fig. 1A, B). Mice treated with IF had smaller hematoma than ICH groups at day 3 and 7 after ICH induction (Fig. 1C, D). Consistently, IF reduced brain edema significantly as indicated by hemisphere volumes at day 3 and 7 except for day 1 (Additional file 1: Table S3, Fig. 1C, E). At day 28, IF-treated mice showed reduced striatum atrophy as indicated by DARPP-32 positive area shrinkage as compared to ICH groups (24.76% ± 5.17% *vs* 18.34% ± 3.59%, n = 6, *p* < 0.05, Fig. 1F, G). TUNEL staining showed that the number of apoptotic cells was reduced at day 1, 3 and 7 (Additional file 1: Table S4, Fig. 1H, I). To further explore neuronal apoptosis, immunofluorescence staining and confocal microscopy for cleaved caspase-3 and the neuron marker NeuN was performed. The data demonstrated that IF reduced apoptotic neuron at day 3 and 7 (Additional file 1: Fig. S2). On the other side, IF preserved the number of Nissl-positive neurons in the ipsilateral hemisphere significantly from day 1 to 28 after ICH (Additional file 1: Table S5, Fig. 1J, K). These results indicated that IF had protective effects against brain damage and neurological deficits in both the acute and chronic stages of ICH in mice.

### IF regulates microglia activation and inflammatory cytokines release after ICH

**Fig. 2:**
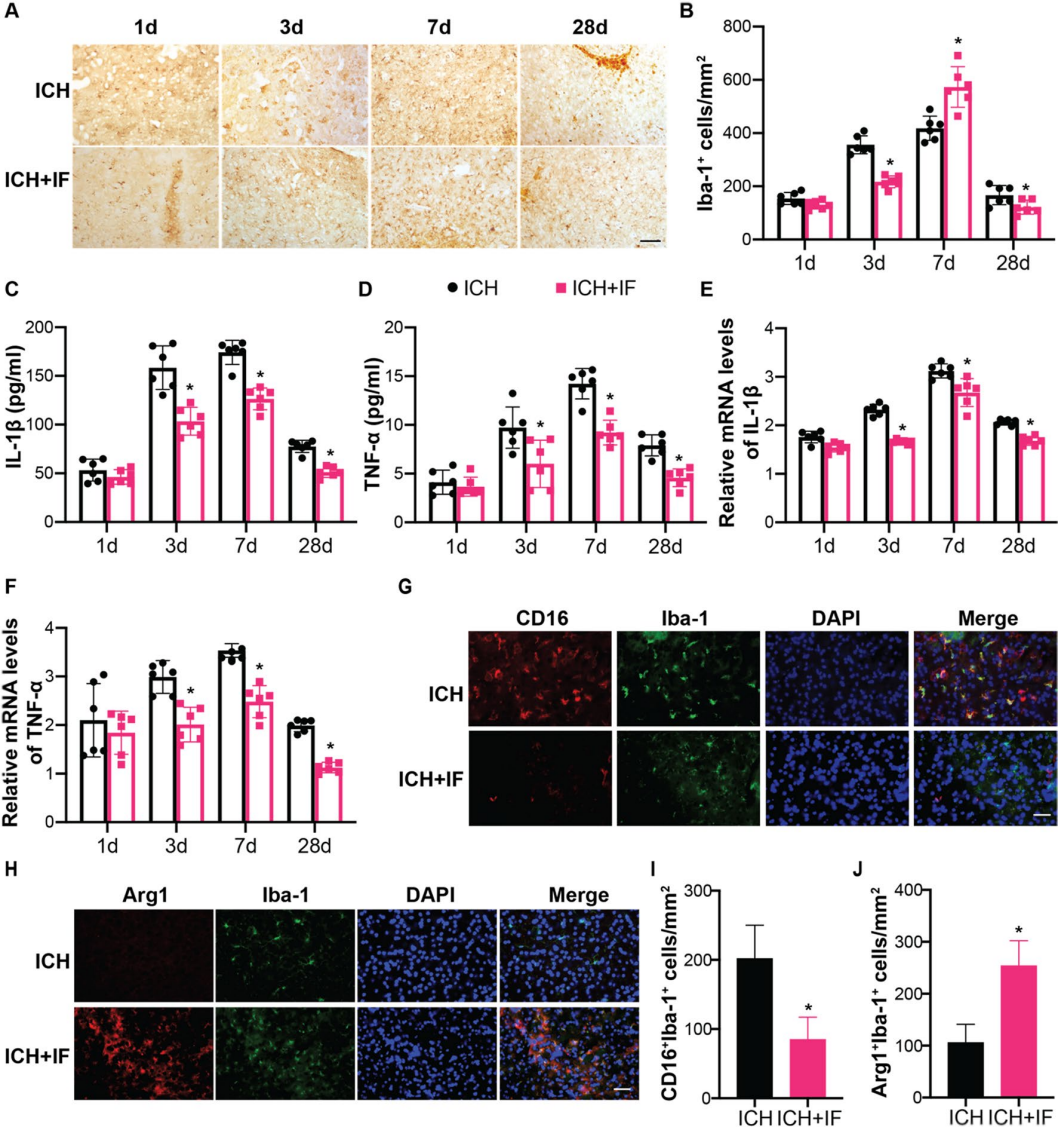
Effects of IF on microglia activation and inflammatory factors release after ICH. **A** Examples of Iba-1 immunohistochemistry in the ipsilateral basal ganglia at different timepoints after ICH, Scale bar = 50 μm. **B** The number of Iba-1^+^ cells was quantified as shown in the bar graphs (*n* = 6). Secretion of IL-1β **C** and TNF-α **D** in blood after ICH was measured by ELISA (*n* = 6). mRNA levels of IL-1β **E** and TNF-α **F** in microglia isolated from ipsilateral cerebra were measured by qRT-PCR. Double fluorescent staining of CD16 (**G**, Red) or Arg1 (**H**, Red) with Iba-1 (Green) in the ipsilateral basal ganglia respectively (Scale bar = 20 μm) and counting of CD16^+^Iba-1^+^ or Arg1^+^Iba-1^+^ cell numbers (*n* = 6). Statistical analysis was performed using one-way ANOVA followed by post hoc Tukey’s test for multiple comparisons (**B**–**F**), Student *t* test between two groups (**I**, **J**). Values are mean ± SD, *P < 0.05 compared with ICH group

We next investigated the effects of IF on microglia activation after ICH. Iba-1 positive microglia accumulated at day 1 in the ipsilateral hemisphere, increased from day 3 to 7, but decreased at day 28 (Fig. 2A, B). Interestingly, the number of Iba-1^+^ microglia and the protein levels of Iba-1 decreased after IF treatment at day 3 and 28 but increased at day 7 (Additional file 1: Tab S6, Fig. 2A, B, Additional file 1: Fig. S3). However, microglia-driven inflammatory response measured with ELISA indicated that proinflammatory factors IL-1β and TNF-α in the blood were increased in response to ICH, which were attenuated by IF-treatment from day 3 to day 28 (Additional file 1: Table S7, Fig. 2C, D). In addition, the mRNA levels of IL-1β and TNF-α in brain tissue around hematoma also increased after ICH, which was attenuated by IF (Additional file 1: Table S8, Fig. 2E, F). Considering the plasticity of microglia in response to different microenvironment, we speculated that the inconsistency in changes of microglia activation and proinflammatory factors secretory was possibly due to different functional types of microglia. As shown in Fig. 2G–J, the proinflammatory marker CD16 and anti-inflammatory marker Arg1 were double stained with Iba-1 at day 3 and 7, respectively. IF significantly reduced the CD16^+^Iba-1^+^ cells at day 3 (202.00 ± 48.00 *vs* 85.00 ± 32, n = 12, *p* < 0.01, Fig. 2G, I) and increased Arg1^+^Iba-1^+^ at day 7 (106.00 ± 35.00 *vs* 254.00 ± 48.00, n = 12, *p* < 0.01, Fig. 1H, J) after ICH onset. These results indicated that IF might suppress microglia-induced inflammation in acute stage and drive microglia polarization towards anti-inflammation phenotype in subacute stage of ICH.

### Microglial Sirt3 is upregulated by IF after ICH

**Fig. 3:**
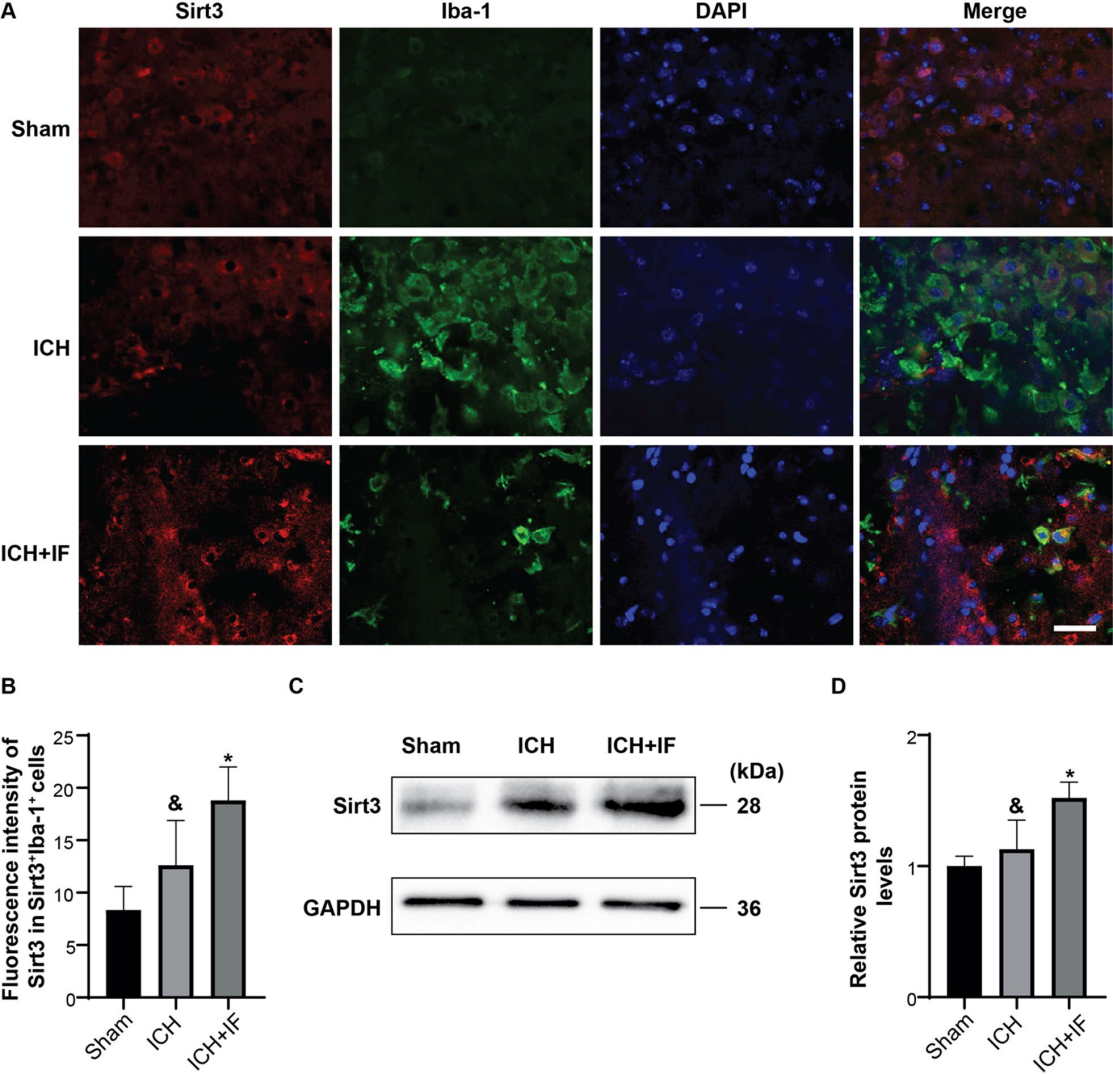
Sirt3 expression in microglia after IF treatment for 3 days in ICH mice. **A** Double fluorescent staining of Sirt3 (Red) and Iba-1 (Green) in the ipsilateral basal ganglia, Scale bar = 20 μm. **B** Evaluation of fluorescence intensity of Sirt3 in the Sirt3^+^Iba-1^+^ cells (*n* = 6). **C** Western blot analysis of Sirt3 protein levels in microglia isolated from ipsilateral basal ganglia and quantification of relative gray value **D** compared with Sham group (*n* = 3). Statistical analysis was performed using one-way ANOVA followed by post hoc Tukey’s test for multiple comparisons. Values are mean ± SD, ^&^*P* < 0.05 compared with Sham group, *P < 0.05 compared with ICH group

We further investigated whether Sirt3 is involved in the effects of IF on microglia activation after ICH. Immunofluorescence staining was used to offer direct evidence of Sirt3 expression in microglia. As shown in Fig. 3A, B, Sirt3 expression was increased in microglia at 3 days after ICH as compared to Sham groups, which further increased in ICH + IF group (sham 8.35 ± 2.24 vs. ICH 12.62 ± 4.26 vs. ICH + IF 18.81 ± 3.17, *n* = 12, *p* < 0.01). Similar results were also obtained for day 7 (ICH 12.45 ± 8.76 vs. ICH + IF 21.23 ± 6.33, *n* = 12, *p* < 0.05, Additional file 1: Fig S4). In addition, western blot also confirmed the upregulated Sirt3 in ICH and ICH + IF groups (Sham 1.00 ± 0.08 *vs* ICH 1.13 ± 0.22 vs. ICH + IF 1.52 ± 0.12, *n* = 5, *p* < 0.01, Fig. 3C, D). These data indicated that Sirt3 in microglia was upregulated by IF, and might play a role in IF-induced neuroprotection after ICH.

### Microglial Sirt3 deletion attenuates the neuroprotective effects of IF following ICH

**Fig. 4:**
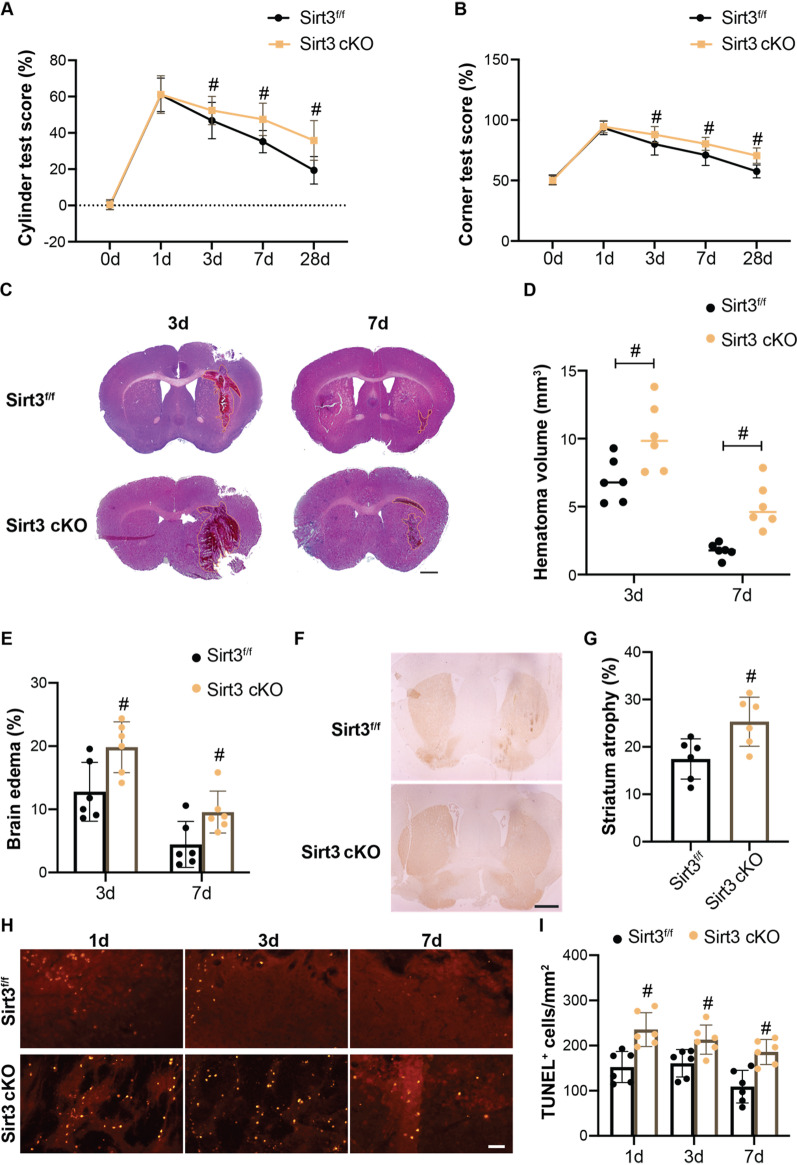
Role of microglial Sirt3 in IF-induced neuroprotective effects after ICH. Cylinder test **A** and corner test **B** tests were performed pre-ICH (*n* = 41) and at day 1 (*n* = 38), 3 (*n* = 27), 7 (*n* = 18) and 28 (*n* = 9) after ICH in Sirt3^f/f^ or Sirt3^f/f^Cx3cr1-Cre mice with IF treatment. **C** Representative HE staining images of the largest clot at day 3 and 7 days after ICH with IF treatment, Scale bar = 1 mm. Quantification of hematoma size **D** and brain volume **E** in Sirt3^f/f^ or Sirt3^f/f^Cx3cr1-Cre mice after ICH with IF (*n* = 6). **F** Examples of DARPP-1 immunohistochemistry in the basal ganglia at 28 day after ICH with IF treatment, Scale bar = 1 mm. **G** Quantification of ipsilateral shrinkage of the DARPP-32 positive area in the mice (*n* = 6). **H** TUNEL staining of ipsilateral mice brain at day 1, 3 and 7 after ICH with IF treatment and **I** the assessment of the number of TUNEL positive cells (*n* = 6), Scale bar = 50 μm. Statistical analysis was performed using two-way ANOVA analysis plus post hoc Bonferroni’s test for behavioral data (**A**, **B**), one-way ANOVA followed by post hoc Tukey’s test for multiple comparisons (**D**, **E**), Student *t* test between two groups (**G**, **I**). Values are mean ± SD, ^#^*P* < 0.05 compared with Sirt3^f/f^ group

Sirt3^f/f^;Cx3cr1-Cre (Sirt3 cKO) mice in which Sirt3 was deleted specifically in microglia were used to determine the role of microglial Sirt3 in IF-induced neuroprotective response. As shown in Fig. 4A, B, Sirt3 cKO mice exhibited worse neurological functions as indicated by the higher corner test and cylinder test scores as compared to control Sirt3^f/f^ mice in both the acute and chronic stages after ICH + IF (Additional file 1: Tables S1, S2). In addition, Sirt3 cKO mice had larger hematoma volume and edema content (Additional file 1: Table S3, Fig. 4C–E) at day 3 and 7 and more severe striatum atrophy at day 28 as compared to control Sirt3^f/f^ mice after ICH + IF (Sirt3^f/f^ 17.45% ± 4.26% *vs* Sirt3 cKO 25.32% ± 5.18%, *n* = 6, *p* < 0.05, Fig. 4F, G). TUNEL staining showed that at day 1 after ICH, the number of TUNEL-positive cells was higher in Sirt3 cKO group (Additional file 1: Table S4, Fig. 4H, I). Besides, condition knockout of Sirt3 in microglia also increased the apoptotic neurons at day 3 and 7 (Additional file 1: Fig. S5). These data suggested that microglial Sirt3 is required for IF-induced neuroprotective effects after ICH.

### Sirt3 contributes to anti-neuroinflammatory effects of IF in the acute stage of ICH

**Fig. 5:**
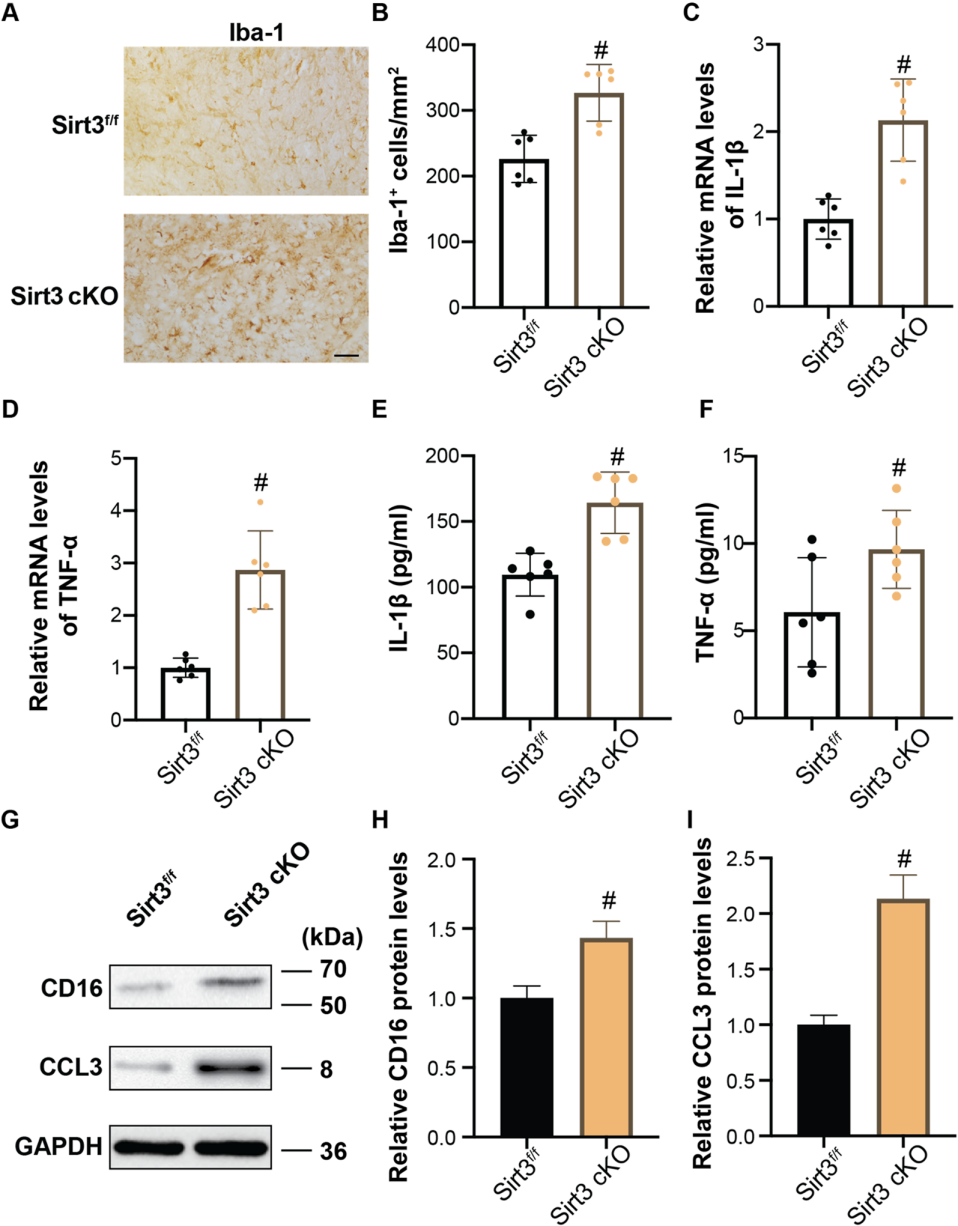
Role of microglial Sirt3 in anti-inflammatory effects of IF at day 3 after ICH. **A** Examples of Iba-1 immunohistochemistry in the ipsilateral basal ganglia ICH, Scale bar = 50 μm. **B** The number of Iba-1^+^ cells was quantified as shown in the bar graphs. mRNA levels of IL-1β **C** and TNF-α **D** in microglia isolated from ipsilateral cerebra were measured by qRT-PCR. Secretion of IL-1β **E** and TNF-α **F** in blood after ICH was measured by ELISA. **G** Western blot analysis of CD16 and CCL3 protein levels in microglia isolated from ipsilateral basal ganglia and quantification of relative gray value of CD16 **H** and CCL3 **I** protein bands. Statistical analysis was performed using Student *t* test. Values are mean ± SD, ^#^*P* < 0.05 compared with Sirt3^f/f^ group

The role of Sirt3 in regulating microglia-driven inflammatory response in ICH + IF in the acute stage of ICH (day 3) was further investigated. At day 3 after ICH + IF, the number of Iba-1^+^ microglia in the perihematomal area was greater in Sirt3 cKO than in Sirt3^f/f^ mice ( Sirt3^f/f^ 226.00 ± 36.00 *vs* Sirt3 cKO 327.00 ± 43.00, n = 6, *p* < 0.01, Fig. 5A, B). Accordingly, the mRNA levels of IL-1β (Sirt3^f/f^ 1.00 ± 0.23 *vs* Sirt3 cKO 2.13 ± 0.47, n = 3, *p* < 0.05, Fig. 5C) and TNF-α (Sirt3^f/f^ 1.00 ± 0.18 *vs* Sirt3 cKO 2.87 ± 0.75, n = 3, *p* < 0.05, Fig. 5D) in perihematomal areas were higher in Sirt3 cKO mice than in Sirt3^f/f^ counterparts. ELISA analysis of the serum also showed higher levels of IL-1β (Sirt3^f/f^ 109.52 ± 16.28 vs. Sirt3 cKO 164.33 ± 23.32 pg/ml, *n* = 6, *p* < 0.01, Fig. 5E) and TNF-α (Sirt3^f/f^ 6.06 ± 3.12 *vs* Sirt3 cKO 9.67 ± 2.23 pg/ml, *n* = 6, *p* < 0.05, Fig. 5F) in Sirt3 cKO mice. Western blot also showed that the proinflammatory markers CD16 (Sirt3^f/f^ 1.00 ± 0.09 vs. Sirt3 cKO 1.43 ± 0.12, *n* = 3, *p* < 0.01, Fig. 5G, H) and CCL3 (Sirt3^f/f^ 1.00 ± 0.09 vs. Sirt3 cKO 2.13 ± 0.21, *n* = 3, *p* < 0.01, Fig. 5G, I) were significantly increased in microglia isolated from perihematomal regions in Sirt3 cKO mice as compared to Sirt3^f/f^ mice.

### Microglial Sirt3 promotes microglia polarization to anti-neuroinflammatory phenotype in the subacute stage of ICH

**Fig. 6:**
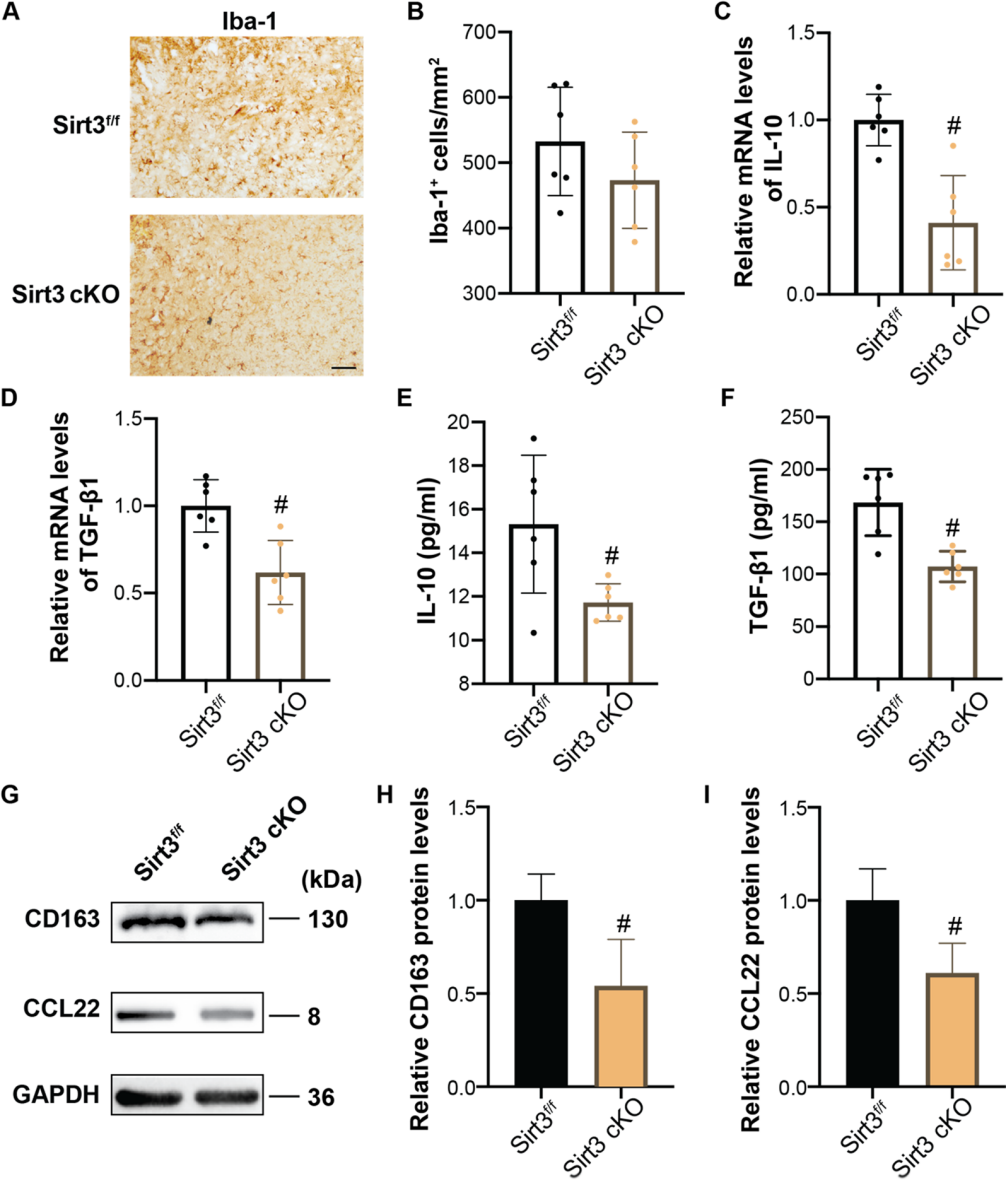
Role of microglial Sirt3 in IF-induced microglia polarization at day 7 after ICH. **A** Examples of Iba-1 immunohistochemistry in the ipsilateral basal ganglia ICH, Scale bar = 50 μm. **B** The number of Iba-1^+^ cells was quantified as shown in the bar graphs. mRNA levels of IL-10 **C** and TGF-β1 **D** in microglia isolated from ipsilateral cerebra were measured by qRT-PCR. Secretion of IL-10 **E** and TGF-β1 **F** in blood after ICH was measured by ELISA. **G** Western blot analysis of CD163 and CCL22 protein levels in microglia isolated from ipsilateral basal ganglia and quantification of relative gray value of CD163 **H** and CCL22 **I** protein bands. Statistical analysis was performed using Student *t* test. Values are mean ± SD, ^#^*P* < 0.05 compared with Sirt3^f/f^ group

Because IF increased Arg^+^Iba-1^+^ cells while reduced proinflammatory factor release in the subacute phase (day 7) after ICH, there parameters were further measured in Sirt3 cKO mice. The number of Iba-1^+^ microglia had limited change in the perihematomal area was not different between Sirt3^f/f^ mice and Sirt3^f/f^ cKO mice at day 7 treated with IF after ICH (Sirt3^f/f^ 532.00 ± 83.00 *vs* Sirt3 cKO 473.00 ± 73.00, *n* = 6, *p* = 0.22, Fig. 6A, B). However, the mRNA levels of IL-10 (Sirt3^f/f^ 1.00 ± 0.15 *vs* Sirt3 cKO 0.41 ± 0.27, *n* = 3, *p* < 0.05, Fig. 6C) and TGF-β1 (Sirt3^f/f^ 1.00 ± 0.15 vs. Sirt3 cKO 0.62 ± 0.18, *n* = 3, *p* < 0.05, Fig. 6D) in perihematomal areas were lower in Sirt3 cKO mice than in Sirt3^f/f^ mice. Consistently, levels of IL-10 (Sirt3^f/f^ 15.32 ± 3.16 *vs* Sirt3 cKO 11.73 ± 0.86, *n* = 6, *p* < 0.01, Fig. 6E) and TGF-β1 (Sirt3^f/f^ 168.44 ± 31.70 vs. Sirt3 cKO 107.23 ± 14.52, *n* = 6, *p* < 0.01, Fig. 6F) in serum also decreased in Sirt3 cKO mice. In addition, the anti-inflammatory markers CD163 (Sirt3^f/f^ 1.00 ± 0.14 vs. Sirt3 cKO 0.54 ± 0.25, *n* = 3, *p* < 0.05, Fig. 6G, H) and CCL22 (Sirt3^f/f^ 1.00 ± 0.17 vs. Sirt3 cKO 0.61 ± 0.16, *n* = 3, *p* < 0.05, Fig. 6G, I) were significantly downregulated in microglia in the perihematomal regions in Sirt3 cKO mice as compared to Sirt3^f/f^ mice. These observations suggest that microglial Sirt3 is involved in the regulation of microglia polarization in ICH + IF.

### Sirt3 regulates microglial activation through Nrf2/HO-1 pathway in acute stage of ICH

**Fig. 7:**
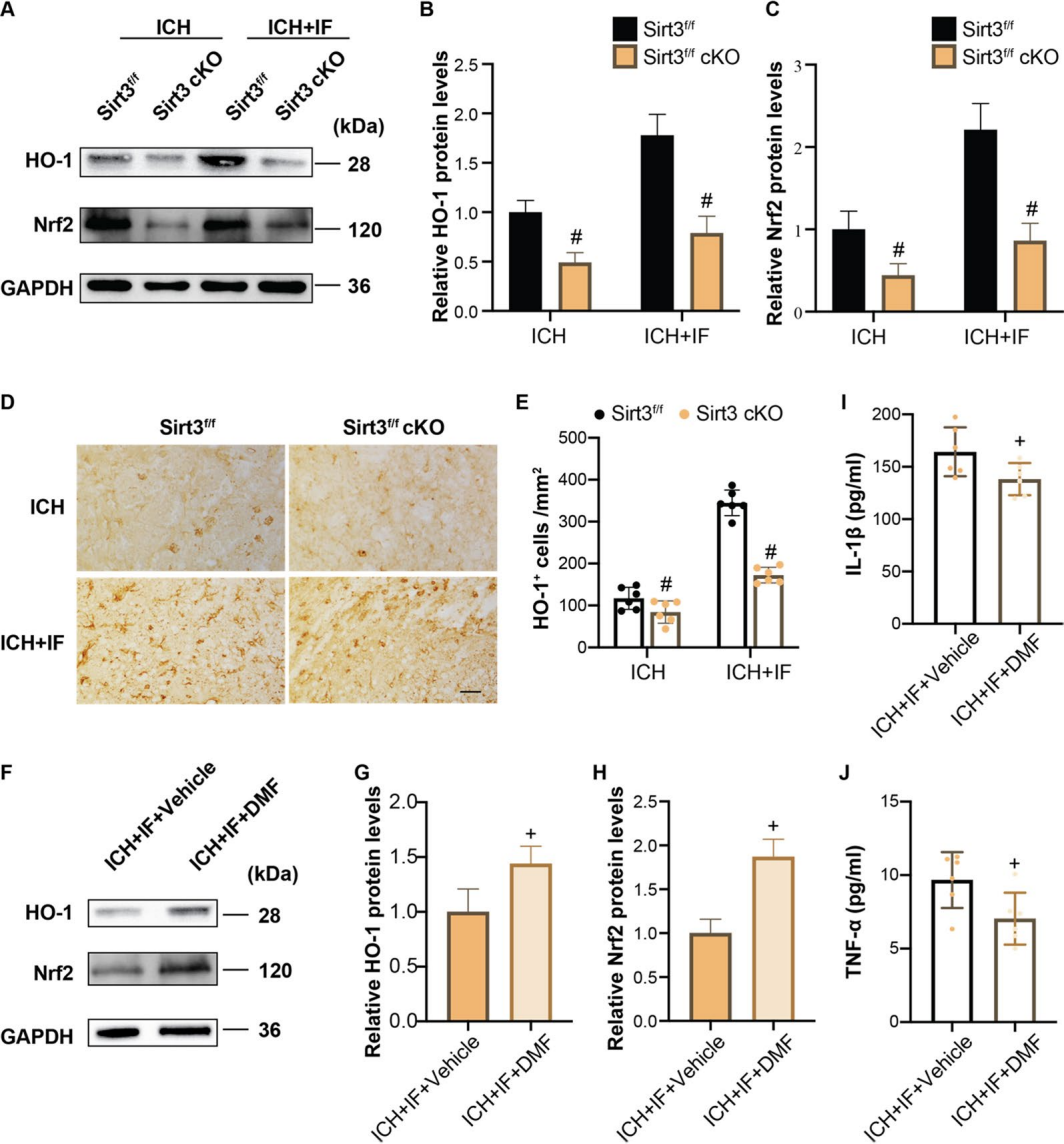
Effects of Nrf2/HO-1 signaling pathways in IF-regulated microglia activation after ICH in Sirt3 cKO mice. **A** Western blot analysis of HO-1 and Nrf2 protein levels in microglia of ipsilateral basal ganglia of Sirt3^f/f^ or Sirt3^f/f^ cKO mice after ICH treated with or without and quantification of relative expression of HO-1 **B** and Nrf2 (**C**). **D** Examples of HO-1 immunohistochemistry in the ipsilateral basal ganglia of Sirt3^f/f^ or Sirt3^f/f^ cKO mice after ICH with or without IF treatment, Scale bar = 50 μm. **E** HO-1^+^ cells were quantitated in the bar graph (*n* = 6). **F** Western blot analysis of HO-1 and Nrf2 protein levels in microglia of ipsilateral basal ganglia and quantification of relative expression of HO-1 **G** and Nrf2 (**H**). Quantification of IL-1β **I** and TNF-α **J** in blood after ICH treated with IF plus vehicle control or DMF. Statistical analysis was performed using one-way ANOVA followed by post hoc Tukey’s test for multiple comparisons (**B**, **C**, **E**), Student *t* test between two groups (**I**–**J**). Values are mean ± SD, ^#^*P* < 0.05 compared with Sirt3^f/f^ group, ^+^*P* < 0.05 compared with ICH + IF + Vehicle group

To investigate whether the Nrf2/HO-1 signaling pathway was regulated by Sirt3 in ICH + IF, the protein levels of Nrf2 and HO-1 were first measured at multiple timepoints in the acute and subacute stages of ICH in WT mice. As shown in Additional file 1: Fig. S6, both Nrf2 and HO-1 proteins increased significantly at day 1 and 3 but decreased almost to the baseline levels at day 7. Therefore, we chose day 3 as the timepoint for further study. At day 3 after ICH, IF upregulated the expressions of Nrf2 and HO-1 in microglia which were attenuated by Sirt3 deletion (Fig. 7A–C). Consistently, IF-induced increases in number of HO-1^+^ cells were attenuated by Sirt3 deletion (Fig. 7D, E). Next, Sirt3^f/f^ cKO mice were pretreated with a clinically approved Nrf2 activator, dimethyl fumarate (DMF) or vehicle control for 7 days before ICH + IF. HO-1 (1.00 ± 0.21 *vs* 1.44 ± 0.16, *n* = 3, *p* < 0.05, Fig. 7F, G) and Nrf2 (1.00 ± 0.16 *vs* 1.87 ± 0.20, *n* = 3, *p* < 0.05, Fig. 6F, H) levels were increased markedly in the ICH + IF + DMF groups as compared to ICH + IF + Vehicle groups. Moreover, the secretion of IL-1β (164.33 ± 23.32 vs. 138.25 ± 15.33, *n* = 6, *p* < 0.05, Fig. 7I) and TNF-α (9.67 ± 1.90 vs. 7.04 ± 1.76, *n* = 6, *p* < 0.05, Fig. 7J) in serum were partly reduced by DMF. To determine whether DMF could recapitulate the beneficial effects of IF in neurological performance and hematoma resolution, neurofunctional scores and clot volume were investigated at day 3 after ICH + IF with DMF pretreatment. The data indicated that DMF significantly improved neurological deficits and reduced hematoma volume (Additional file 1: Fig. S7). Altogether, these results indicated that the Nrf2/HO-1 signaling pathway may be downstream of Sirt3 in microglial activation at day 3 after ICH.

## Discussion

Inflammation caused by degradation products of red blood cells (RBCs) following intracerebral hemorrhage leads to severe secondary brain injury, which is a major reason leading to neuronal apoptosis and worsening prognosis of ICH [24]. However, there is no effective clinical intervention so far. The present study demonstrated for the first that intermittent fasting protected against ICH-induced neurologic deficits in both short and long terms by suppressing microglia-driven inflammatory response and neuronal apoptosis. Notably, genetic deletion of microglia Sirt3 abolished the beneficial effects of IF through inhibiting Nrf2/HO-1 signaling pathway in the acute stage after ICH. These results suggested a critical role of Sirt3 in ICH-induced brain injury, especially in regulating neuroinflammatory responses after ICH.

Since Goodrick et al. first demonstrated that intermittent fasting extends mice lifespan significantly [25], subsequent studies showed that IF also has multiple benefits in the management of cardiovascular disease, hypertension, liver ischemia, metabolic disorders, obesity and adiposity through decreasing metabolic homeostasis, oxidative stress and inflammation [26, 27]. In CNS disorders, IF has been shown to improve cognitive performance in Alzheimer’s disease, Huntington’s disease and neuropsychiatric diseases [14, 28]. A recent study supports the notion that IF alleviates poststroke brain damage via altering epigenetic and transcriptional programming [17]. In the present study, we have explored whether IF has any effects on ICH-induced brain damage. Our results indicates that every-other-day feed strategy ameliorates ICH-induced neurological functional deficits in mice at day 3–28. The protective effects of IF may be partly through enhancing hematoma clearance and reducing brain edema after ICH. Furthermore, the release of proinflammatory cytokines, such as IL-1β and TNFα was suppressed by IF treatment in the multiple phases of ICH, strongly supporting the essential role of inflammation in functional recovery after ICH, which may be the underlying mechanism contributed to the neuroprotective effects of IF. Interestingly, the beneficial effects of IF were limited at day 1 in our study. The underlying reason maybe that it doesn’t work directly on the brain but through a systematic response. A body of literature has shown that intermittent fasting or calorie restriction works by regulating energetic metabolism 12 to 24 h later after depletion of carbohydrates [26]. Besides, IF has been proved to effectively restored the gut microbiome, and then probably influences neurological function [29], which is an increasingly popular research topic in recent years. Therefore, intermittent fasting probably protect brain from ICH insult through shifting energetic metabolism after a certain time. However, whether this assumption holds still requires further investigation.

Microglia are known as the main innate immune cells in CNS which evoke inflammatory mediator release when sense danger signals after ICH insult [8]. Increasing evidence has indicated that the inhibition of activated microglia ameliorate neuronal injuries after ICH [30]. Consistent with the previous studies, we find the potential link between downregulated Iba-1^+^ microglia number by IF and improved neurological function at day 3 and 28 after ICH. However, the other major function of microglia is to engulf RBCs and remove cellular debris [31]. Under certain conditions, microglia can also express arginase1 (Arg1), insulin-like growth factor (IGF-1), Ym1 and anti-inflammatory cytokines, such as IL-10 and IL-4, which facilitate recovery of CNS injury including ischemic stroke [32]. Considering the high plasticity of microglia according to different pathologic events or microenvironments [33], deeper insight into the regulatory mechanisms of microglial phenotype variations may help maintain the homeostasis of microglia activation. Intriguingly, in the present study, IF increased Iba-1^+^ microglia at day 7 after ICH while supressed proinflammatory cytokine release. To gain an initial understanding of the characteristics of the increased microglia, we used the fluorescence double-labeling and found most of these microglia expressed the anti-inflammatory marker Arg1. It is possible that IF promotes microglia polarize to the anti-inflammatory subtype at the subacute stage after ICH. Besides, although IF decreased the number of Iba-1^+^ microglia at the acute stage, the hematoma clearance was enhanced. The clearance of hematoma may depend on some unknown cells other than activated microglia which still need further investigations.

The underlying mechanisms of IF in regulating inflammation after ICH are still unknown. It has been reported that energy deficiency caused by IF leads metabolic changes to oxidative phosphorylation and maintain stable mitochondrial function [16, 34]. In these processes, Sirtuin family proteins probably have a crucial role. Sirtuin-3 (Sirt3) is one of the important and extensively studied proteins of Sirutins which locates mainly in mitochondria and regulates mitochondrial functions and energy metabolism in a variety of physiological and pathological processes [35]. To uncover whether Sirt3 participates in the IF-induced anti-inflammatory functions, we first analysed the protein levels of Sirt3 in microglia treated with IF and found the potential positive effects on regulating Sirt3 expression by IF. A conditional microglia-specific Sirt3 depletion mouse strain is further applied to explore its effects under the condition of energy restriction after ICH. Mice lacking Sirt3 in microglia have poor neurological functional retention in both short and long terms treated with IF. In addition, the proinflammatory markers, such as CD16, CCL3, IL-1β and TNF-α increased in Sirt3^f/f^Cx3cr1-Cre mice compared with Sirt3^f/f^ mice at day 3 after ICH, while the anti-inflammatory markers decreased at day7, suggesting that IF may act through microglial Sirt3 to manipulate neuroinflammation after ICH. It is worth noting, though, Cx3cr1-driven Cre recombinase is a widely used genetic tool for enabling gene manipulation in microglia [36, 37], nonmicroglial cells expressing Cx3cr1, such as macrophage and leakiness of cre activity into neurons have become a major concern [38]. In the present study, conditional knockout of Sirt3 in cells with Cx3cr1 promoter attenuated the protective effects of IF. It is reasonable to get the view that Sirt3 in microglia participates in the effects of IF, while there may be other cells included since it is hardly to distinguish nonmicroglia, which was a limitation of our work.

As reported, Nrf2/HO-1 signaling pathway has been proved to function as one of the key molecular mechanisms participating in oxidative stress and inflammatory activity in multiple organs and cells including colon, kidney and brain [39–41]. Under the condition of dietary energy restriction, Nrf2 activity can be reinforced therefore regulates the transcription, modification, or expression of downstream proteins, such as HO-1, glutathione (GSH), superoxide dismutase (SOD) and fibroblast growth factor (FGF) [42, 43]. It has been previously reported that in mice AD model, IF has the beneficial effects on anxiety and cognition which is mediated by Sirt3 through increasing the enzymatic activity of SOD [28]. Correspondently, in Sirt3 null mice, the application of Nrf2 agonist DMF can suppress the release of IL-1β and TNF-α as well as increase HO-1 expression at a certain level although not to the baseline of WT mice. These finding illustrates that Nrf2/HO-1 pathway may be at least partly associated with neurological adaptations and inflammatory inhibition by IF after ICH.

## Conclusions

**Fig. 8:**
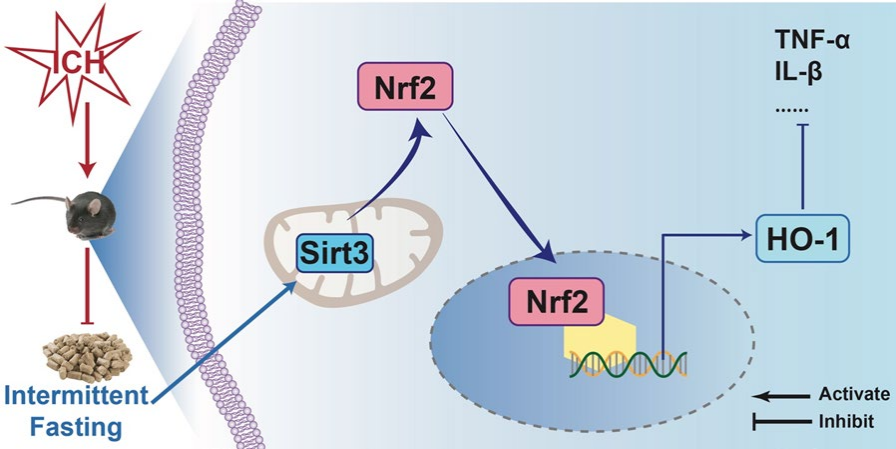
IF alleviated neurological deficits and supressed neuroinflammation after ICH via Sirt3/Nrf2/HO-1 pathway

In conclusion, our results revealed that IF limits neurological deficits and neuronal apoptosis in early ICH injury, and has a long-term protective effects against neuroinflammation. The neuroprotective effects of IF are mediated at least partly through Sirt3/Nrf2/HO-1 signaling pathways in microglia (Fig. 8). However, the possible link in the neuronal network between microglia and neurons under the above condition still need further study. Overall, IF may considerably serve as a potential nonpharmacological dietary paradigm treating ICH.

## Supplementary Information

**Additional file 1. **Additional materials, tables and figures.

## Data Availability

Data for all graphs in this article were available in the Additional file 1: Data Supplement except listed above.

## Abbreviations

ICH: Intracerebral hemorrhage
IF: Intermittent fasting
Iba-1: Ionized calcium-binding adaptor molecule 1
DARPP-32: Dopamine and adenosine 3′5'-monophosphate-regulated phospho-protein
HO-1: Heme oxygenase 1
HE: Hematoxylin–eosin
Arg1: Arginase1
Sirt3: Sirtuin-3
IL: Interleukin
TUNEL: TdT-mediated dUTP nick end labeling
iNOS: Inducible nitric oxide synthase
TNF-α: Tumor necrosis factor-α
CNS: Central nervous system
Nrf: Nuclear respiratory factor
NADH: Nicotinamide adenine dinucleotide
WT: Wild type
BSA: Bovine serum albumin
PBS: Phosphate balanced solution
DAPI: 4,6-Diamidino-2-phenylindole
DAB: 3,3’-Diaminobenzidine
ELISA: Enzyme-linked immunosorbent assay
SD: Standard deviation
DMF: Dimethyl fumarate
RBCs: Red blood cells
IGF-1: Insulin-like growth factor
GSH: Glutathione
SOD: Superoxide dismutase
FGF: Fibroblast growth factor

## References

[1] Li Z, Li M, Shi SX, Yao N, Cheng X, Guo A (2020). Brain transforms natural killer cells that exacerbate brain edema after intracerebral hemorrhage. J Exp Med.

[2] Vaibhav K, Braun M, Khan MB, Fatima S, Saad N, Shankar A (2018). Remote ischemic post-conditioning promotes hematoma resolution via ampk-dependent immune regulation. J Exp Med.

[3] Mendelow AD, Gregson BA, Rowan EN, Murray GD, Gholkar A, Mitchell PM (2013). Early surgery versus initial conservative treatment in patients with spontaneous supratentorial lobar intracerebral haematomas (stich ii): a randomised trial. Lancet.

[4] Kuramatsu JB, Biffi A, Gerner ST, Sembill JA, Sprugel MI, Leasure A (2019). Association of surgical hematoma evacuation vs conservative treatment with functional outcome in patients with cerebellar intracerebral hemorrhage. JAMA.

[5] Zhao X, Ting SM, Liu CH, Sun G, Kruzel M, Roy-O'Reilly M (2017). Neutrophil polarization by il-27 as a therapeutic target for intracerebral hemorrhage. Nat Commun.

[6] Tao C, Keep RF, Xi G, Hua Y (2020). Cd47 blocking antibody accelerates hematoma clearance after intracerebral hemorrhage in aged rats. Transl Stroke Res.

[7] Zhang Z, Zhang Z, Lu H, Yang Q, Wu H, Wang J (2017). Microglial polarization and inflammatory mediators after intracerebral hemorrhage. Mol Neurobiol.

[8] Wang M, Ye X, Hu J, Zhao Q, Lv B, Ma W (2020). Nod1/rip2 signalling enhances the microglia-driven inflammatory response and undergoes crosstalk with inflammatory cytokines to exacerbate brain damage following intracerebral haemorrhage in mice. J Neuroinflammation.

[9] Dai S, Hua Y, Keep RF, Novakovic N, Fei Z, Xi G (2019). Minocycline attenuates brain injury and iron overload after intracerebral hemorrhage in aged female rats. Neurobiol Dis.

[10] Fontana L, Partridge L, Longo VD (2010). Extending healthy life span–from yeast to humans. Science.

[11] Kim C, Pinto AM, Bordoli C, Buckner LP, Kaplan PC, Del Arenal IM (2020). Energy restriction enhances adult hippocampal neurogenesis-associated memory after four weeks in an adult human population with central obesity; a randomized controlled trial. Nutrients.

[12] Liu Z, Dai X, Zhang H, Shi R, Hui Y, Jin X (2020). Gut microbiota mediates intermittent-fasting alleviation of diabetes-induced cognitive impairment. Nat Commun.

[13] Witte AV, Fobker M, Gellner R, Knecht S, Floel A (2009). Caloric restriction improves memory in elderly humans. Proc Natl Acad Sci U S A.

[14] Dias GP, Murphy T, Stangl D, Ahmet S, Morisse B, Nix A (2021). Intermittent fasting enhances long-term memory consolidation, adult hippocampal neurogenesis, and expression of longevity gene klotho. Mol Psychiatry..

[15] Matsui S, Sasaki T, Kohno D, Yaku K, Inutsuka A, Yokota-Hashimoto H (2018). Neuronal sirt1 regulates macronutrient-based diet selection through fgf21 and oxytocin signalling in mice. Nat Commun.

[16] Longo VD, Cortellino S (2020). Fasting, dietary restriction, and immunosenescence. J Allergy Clin Immunol.

[17] Vemuganti R, Arumugam TV (2021). Much ado about eating: Intermittent fasting and post-stroke neuroprotection. J Cereb Blood Flow Metab.

[18] Stagg DB, Gillingham JR, Nelson AB, Lengfeld JE, Andre d'Avignon D, Puchalska P (2021). Diminished ketone interconversion, hepatic tca cycle flux, and glucose production in d-beta-hydroxybutyrate dehydrogenase hepatocyte-deficient mice. Mol Metab.

[19] Chen T, Dai SH, Li X, Luo P, Zhu J, Wang YH (2018). Sirt1-sirt3 axis regulates human blood-brain barrier permeability in response to ischemia. Redox Biol.

[20] Wei J, Wang M, Jing C, Keep RF, Hua Y, Xi G (2020). Multinucleated giant cells in experimental intracerebral hemorrhage. Transl Stroke Res.

[21] Chang CF, Massey J, Osherov A, Angenendt da Costa LH, Sansing LH. Bexarotene enhances macrophage erythrophagocytosis and hematoma clearance in experimental intracerebral hemorrhage. Stroke 2020;51:612–618 10.1161/STROKEAHA.119.027037 PMC7135897 31826730

[22] Zhao L, Chen S, Sherchan P, Ding Y, Zhao W, Guo Z (2018). Recombinant ctrp9 administration attenuates neuroinflammation via activating adiponectin receptor 1 after intracerebral hemorrhage in mice. J Neuroinflammation.

[23] Mo J, Enkhjargal B, Travis ZD, Zhou K, Wu P, Zhang G (2019). Ave 0991 attenuates oxidative stress and neuronal apoptosis via mas/pka/creb/ucp-2 pathway after subarachnoid hemorrhage in rats. Redox Biol.

[24] Chang CF, Goods BA, Askenase MH, Hammond MD, Renfroe SC, Steinschneider AF (2018). Erythrocyte efferocytosis modulates macrophages towards recovery after intracerebral hemorrhage. J Clin Invest.

[25] Goodrick CL, Ingram DK, Reynolds MA, Freeman JR, Cider N (1990). Effects of intermittent feeding upon body weight and lifespan in inbred mice: interaction of genotype and age. Mech Ageing Dev.

[26] Touyz RM (2021). Gut dysbiosis-induced hypertension is ameliorated by intermittent fasting. Circ Res.

[27] Miyauchi T, Uchida Y, Kadono K, Hirao H, Kawasoe J, Watanabe T (2019). Up-regulation of foxo1 and reduced inflammation by beta-hydroxybutyric acid are essential diet restriction benefits against liver injury. Proc Natl Acad Sci U S A.

[28] Liu Y, Cheng A, Li YJ, Yang Y, Kishimoto Y, Zhang S (2019). Sirt3 mediates hippocampal synaptic adaptations to intermittent fasting and ameliorates deficits in app mutant mice. Nat Commun.

[29] Varady KA, Cienfuegos S, Ezpeleta M, Gabel K (2021). Cardiometabolic benefits of intermittent fasting. Annu Rev Nutr.

[30] Deng S, Jin P, Sherchan P, Liu S, Cui Y, Huang L (2021). Recombinant ccl17-dependent ccr4 activation alleviates neuroinflammation and neuronal apoptosis through the pi3k/akt/foxo1 signaling pathway after ich in mice. J Neuroinflammation.

[31] Li Q, Lan X, Han X, Durham F, Wan J, Weiland A (2021). Microglia-derived interleukin-10 accelerates post-intracerebral hemorrhage hematoma clearance by regulating cd36. Brain Behav Immun.

[32] Liu Y, Deng S, Song Z, Zhang Q, Guo Y, Yu Y (2021). Mlif modulates microglia polarization in ischemic stroke by targeting eef1a1. Front Pharmacol.

[33] Liu J, Liu L, Wang X, Jiang R, Bai Q, Wang G (2021). Microglia: a double-edged sword in intracerebral hemorrhage from basic mechanisms to clinical research. Front Immunol.

[34] Rojas-Morales P, Leon-Contreras JC, Aparicio-Trejo OE, Reyes-Ocampo JG, Medina-Campos ON, Jimenez-Osorio AS (2019). Fasting reduces oxidative stress, mitochondrial dysfunction and fibrosis induced by renal ischemia-reperfusion injury. Free Radic Biol Med.

[35] Zhou ZD, Tan EK (2020). Oxidized nicotinamide adenine dinucleotide-dependent mitochondrial deacetylase sirtuin-3 as a potential therapeutic target of parkinson's disease. Ageing Res Rev.

[36] Sahasrabuddhe V, Ghosh HS (2022). Cx3cr1-cre induction leads to microglial activation and ifn-1 signaling caused by DNA damage in early postnatal brain. Cell Rep.

[37] Zhou K, Han J, Lund H, Boggavarapu NR, Lauschke VM, Goto S (2022). An overlooked subset of cx3cr1(wt/wt) microglia in the cx3cr1(creer-eyfp/wt) mouse has a repopulation advantage over cx3cr1(creer-eyfp/wt) microglia following microglial depletion. J Neuroinflammation.

[38] Zhang B, Zou J, Han L, Beeler B, Friedman JL, Griffin E (2018). The specificity and role of microglia in epileptogenesis in mouse models of tuberous sclerosis complex. Epilepsia.

[39] Vasconcelos AR, da Paixao AG, Kinoshita PF, Orellana AM, Scavone C, Kawamoto EM (2021). Toll-like receptor 4 signaling is critical for the adaptive cellular stress response effects induced by intermittent fasting in the mouse brain. Neuroscience.

[40] Khan I, Saeed K, Jo MG, Kim MO (2021). 17-beta estradiol rescued immature rat brain against glutamate-induced oxidative stress and neurodegeneration via regulating nrf2/ho-1 and map-kinase signaling pathway. Antioxidants (Basel).

[41] Zhou YQ, Mei W, Tian XB, Tian YK, Liu DQ, Ye DW (2021). The therapeutic potential of nrf2 inducers in chronic pain: evidence from preclinical studies. Pharmacol Ther.

[42] Fang Y, Chen B, Gong AY, Malhotra D, Gupta R, Dworkin LD (2021). The ketone body beta-hydroxybutyrate mitigates the senescence response of glomerular podocytes to diabetic insults. Kidney Int.

[43] Abrescia P, Treppiccione L, Rossi M, Bergamo P (2020). Modulatory role of dietary polyunsaturated fatty acids in nrf2-mediated redox homeostasis. Prog Lipid Res.

