# Supplementary material for: Intermittent fasting reduces neuroinflammation in intracerebral hemorrhage through the Sirt3/Nrf2/HO-1 pathway
Source: J Neuroinflammation. 2022 May 27;19:122. doi: 10.1186/s12974-022-02474-2 (PMC9137193; doi:10.1186/s12974-022-02474-2)
Supplement: Supplementary file 1 — Additional file 1. Additional materials, tables and figures. [file 12974_2022_2474_MOESM1_ESM.docx]

**ADDITIONAL FILE 1**

**Intermittent Fasting Reduces Neuroinflammation in Intracerebral Hemorrhage through the Sirt3/Nrf2/HO-1 Pathway**

**Shuhui Dai,** MD^1,4†^, **Jialiang Wei,** MD^1,2†^, **Hongchen Zhang,** BS^1†^, **Peng Luo,** MD^1^, **Yuefan Yang,** MD^1^, **Xiaofan Jiang,** MD^1^, **Zhou Fei,** MD^1^, **Wenbin Liang,** MD, Ph.D^3^, **Jianli Jiang,** Ph.D^4*^, and **Xia Li,** MD^1*^

^1^Department of Neurosurgery, Xijing Hospital, Fourth Military Medical University, Xi’an, China

^2^Department of Health Service, Fourth Military Medical University, Xi’an, China

^3^University of Ottawa Heart Institute, Department of Cellular and Molecular Medicine, University of Ottawa, Ottawa, ON, Canada

^4^National Translational Science Center for Molecular Medicine and Department of Cell Biology, Fourth Military Medical University, Xi’an, China

†These authors have contributed equally to this work

*Drs. Jianli Jiang and Xia Li shared the last authorship

**Correspondence:**

Xia Li, MD, Department of Neurosurgery, Xijing Hospital, Fourth Military Medical University, 127 Changlexi Rd, Xi’an, China. Email: [lixia_fmmu@163.com](mailto:lixia_fmmu@163.com)

Jianli Jiang, MD, National Translational Science Center for Molecular Medicine and Department of Cell Biology, Fourth Military Medical University, 169 Changlexi Rd, Xi’an, China. Email: [jiangjl@fmmu.edu.cn](mailto:jiangjl@fmmu.edu.cn)

**Expanded Materials and Methods**

*Intracerebral hemorrhage model continued*

Briefly, mice were anesthetized using 5% chloral hydrate (375 mg/kg, i.p.). Autologous blood was extracted with 0.28-diameter polyethylene tubing (Intramedic, USA). 30 μl blood was injected into right basal ganglia at the rate of 3 μl/min (coordinates: 0.2 mm anterior, 2.5 mm lateral and 3.5 mm ventral) with a 1ml syringe. The needle was removed 10 min after injection. During the surgery, a heating pad was used to maintain body temperature.

*Behavioural tests continued*

For corner test, mice were advanced into a 30° corner and allowed to exit by turning either right or left. A total of 20 trails were recorded and corner test score was evaluated as right turns/all trials×100%. For Cylinder test, mice were placed in a transparent glass cylinder of 10-cm in diameter. The numbers of unilateral and bilateral wall contacts of mice were recorded within 3 min. Score was calculated as (right-left)/(right+left+both)×100%. The higher score of either the tests indicated more severe left hemiparesis. All the experiments were performed by experimenters blinded to the identity of groups.

*Nissl and hematoxylin-eosin (H&E) staining*

Nissl staining was performed to meausre the neuronal loss after ICH. The frozen 18-μm-thick brain coronal slices of mice were soaked in Nissl staining solution for 5-15 min and then decolorized in 75, 80, 95, and 100% ethanol for 2 min each. Then the slices were sealed and observed under a microscope. Nissl-positive cells were counted with ImageJ software (version 1.8.0) under 20x magnification. HE staining was used to evaluate the hematoma volume and brain edema indicated by brain volume. The slices with the largest clot were chosen and stained with H&E. All the slices with clot were counted as N. Slicings were scanning with a Leica DM6 microscope and volumes were measured with ImageJ software (version 1.8.0). Hematoma volume was calculated as S_max_×N×0.018/2 μl. Brain edema was calculated as (S_ipsilateral hemisphere_-S_contralateral hemisphere_)/S_contralateral hemisphere_×100%.

*Microglia isolation*

Isolation of microglia from neural cells of mice was performed following the manufacturer’s instruction of Adult Brain Dissociation Kit (Miltenyi Biotec, Germany). In brief, the ipsilateral brain tissue was transferred to a gentleMACS C Tube containing both enzyme mixes. Tissue was enzymaticly dissociated with program 37℃-ABDK-01 of the gentleMACS Octo Dissociator with Heaters (Miltenyi Biotec). Afterwards, the homogenate was applied to the 70 µm cell strainer and the suspension was centrifuged at 4℃ with 300 g for 10 min. Red blood cells and debris were removed via Red Blood Cell Removal Solution at a 1:10 dilution in double-distilled water (ddH_2_O). The remaining cell suspension was incubated with FcR Blocking Reagent and then labelled with CD11b-APC antibody at 4℃ in the dark for 15 min. Microglia-bound antibodies were detected and harvested for the subsequent investigations.

*RT-PCR*

Total mRNA was extracted from isolated microglia using Trizol reagent. RT-PCR was performed in triplicate using the SYBR Green method and the transcriptional level of glyceraldehyde 3-phosphate dehydrogenase (GAPDH) was used as an internal control. mRNA expression levels of candidate genes were calculated using 2^-ΔΔCT^ method. Primers are listed in the Tab S10 below.

*Western blot analysis*

Mice were anesthetized and perfused with ice-cold PBS at different timepoints after ICH. Brain tissue around hematoma was collected for cell and protein extraction. Protein concentration was quantified by BCA method with a commercial kit (Beyotime, China). 20 μg protein of each group was electrophoresed on 10% SDS-PAGE gel and transferred onto polyvinylidene difluoride (PVDF) membrane. The membranes were incubated at 4℃ overnight with primary antibodies diluted in PBS as listed below. Then, the membranes were washed 3 times with PBS and incubated with corresponding secondary antibodies (ABclonal, China) at room temperature for 2 h. Afterwards, the membranes were washed 3 times and bands were detected with enhanced chemiluminescence reagent kit (SuperSignal West Pico, USA). The quantification of target protein densitometry was analysed with ImageJ software (version 1.8.0) and expressed as a relative value normalized to GAPDH levels.

*Primary Antibodies Dilution Ratio*

For immunohistochemistry and immunofluorescence: Sirt3 1:100 (Invitrogen, PA5-115903), HO-1 1:500 (Invitrogen, MA1-112), Iba-1 1:800 (Sigma-Aldrich, MABN92), DARPP-32 1:500 (Cell Signaling Technology, 2306), CD16 1:200 (Abcam, ab246222), Arg1 1:200 (Cell Signaling Technology, 93668), cleaved caspase-3 1:400 (Cell Signaling Technology, 9662), NeuN 1:100 (Millipore, 3612227).

For western blot analysis: Sirt3 1:1000 (Invitrogen, PA5-115903), CD16 1:800 (Abcam, ab246222), CCL3 1:1000 (Invitrogen, MA5-24364), CD163 1:1000 (Invitrogen, PA5-78961), CCL22 1:1000 (Invitrogen, PA5-114960), Nrf2 1:1000 (Invitrogen, PA5-27882), HO-1 1:1000 (Invitrogen, MA1-112), Iba-1 1:800 (Sigma-Aldrich, MABN92), and GAPDH 1:5000 (Abcam, ab8245).

*Data availability*

**Additional file 1 Table 1:** Cylinder test scores (%, data was shwon as mean±SD)

| Groups  Time (day) | WT mice | | Sirt3^f/f^ mice | Sirt3 cKO mice |
| --- | --- | --- | --- | --- |
|  | ICH | ICH+IF | ICH+IF | ICH+IF |
| 0 | -0.06±2.72 | 0.05±2.57 | 0.04±2.29 | 0.40±2.81 |
| 1 | 59.05±11.69 | 57.05±9.16 | 60.98±9.27 | 61.13±10.33 |
| 3 | 55.56±9.46 | 42.62±6.88 | 46.81±9.98 | 52.37±7.79 |
| 7 | 43.26±11.22 | 33.27±6.27 | 35.19±6.13 | 47.44±8.92 |
| 28 | 33.95±10.85 | 17.86±7.97 | 19.39±7.56 | 35.82±10.98 |

**Additional file 1 Table 2:** Corner test scores (%, data was shwon as mean±SD)

| Groups  Time (day) | WT mice | | Sirt3^f/f^ mice | Sirt3 cKO mice |
| --- | --- | --- | --- | --- |
|  | ICH | ICH+IF | ICH+IF | ICH+IF |
| 0 | 49.76±3.82 | 50.98±3.57 | 50.63±3.95 | 50.12±3.79 |
| 1 | 92.97±5.95 | 92.84±6.30 | 93.47±5.58 | 94.44±5.04 |
| 3 | 87.04±6.69 | 79.82±9.86 | 80.00±8.88 | 87.96±6.54 |
| 7 | 81.39±7.03 | 69.21±7.31 | 71.11±8.67 | 80.31±5.31 |
| 28 | 70.83±5.57 | 58.89±6.97 | 57.50±5.35 | 70.56±6.35 |

**Additional file 1 Table 3:** Brain edema (%, data was shwon as mean±SD)

| Groups  Time (day) | WT mice | | Sirt3^f/f^ mice | Sirt3 cKO mice |
| --- | --- | --- | --- | --- |
|  | ICH | ICH+IF | ICH+IF | ICH+IF |
| 1 | 8.69±2.32 | 7.54±2.65 |  |  |
| 3 | 18.86±4.20 | 13.06±5.51 | 12.78±4.67 | 19.83±4.02 |
| 7 | 10.43±2.82 | 5.19±1.94 | 4.44±3.64 | 9.56±3.31 |

**Additional file 1 Table 4:** TUNEL^+^ cells (/mm^2^, data was shwon as mean±SD)

| Groups  Time (day) | WT mice | | Sirt3^f/f^ mice | Sirt3 cKO mice |
| --- | --- | --- | --- | --- |
|  | ICH | ICH+IF | ICH+IF | ICH+IF |
| 1 | 228.00±31.00 | 176.00±28.00 | 152.00±34.00 | 235.00±37.00 |
| 3 | 212.00±23.00 | 156.00±19.00 | 161.00±30.00 | 213.00±32.00 |
| 7 | 174.00±34.00 | 117.00±22.00 | 109.00±36.00 | 186.00±28.00 |

**Additional file 1 Table 5:** Nissl^+^ cells (/mm^2^, data was shwon as mean±SD)

| Time (day)  Groups | 1 | 3 | 7 | 28 |
| --- | --- | --- | --- | --- |
| ICH | 842±132 | 1268±117 | 1932±165 | 2013±254 |
| ICH+IF | 1365±208 | 1873±227 | 2434±212 | 2294±166 |

**Additional file 1 Table 6:** Iba-1^+^ cells (/mm^2^, data was shwon as mean±SD)

| Time (day)  Groups | 1 | 3 | 7 | 28 |
| --- | --- | --- | --- | --- |
| ICH | 154±22 | 356±34 | 418±46 | 167±36 |
| ICH+IF | 131±16 | 217±22 | 573±76 | 122±26 |

**Additional file 1 Table 7:** Cytokines release (pg/ml, data was shwon as mean±SD)

| Groups  Time (day) | IL-1β | | TNF-α | |
| --- | --- | --- | --- | --- |
|  | ICH | ICH+IF | ICH | ICH+IF |
| 1 | 53.32±11.23 | 46.33±7.43 | 4.12±1.23 | 3.67±0.98 |
| 3 | 158.54±22.34 | 103.43±14.32 | 9.72±2.12 | 6.01±2.43 |
| 7 | 174.23±12.35 | 126.43±11.24 | 14.23±1.56 | 9.22±1.27 |
| 28 | 77.65±6.32 | 51.22±5.34 | 7.90±1.08 | 4.58±0.92 |

**Additional file 1 Table 8:** mRNA levels (/GAPDH, data was shwon as mean±SD)

| Groups  Time (day) | IL-1β | | TNF-α | |
| --- | --- | --- | --- | --- |
|  | ICH | ICH+IF | ICH | ICH+IF |
| 1 | 1.76±0.12 | 1.54±0.09 | 2.01±0.76 | 1.84±0.44 |
| 3 | 2.32±0.11 | 1.66±0.04 | 2.99±0.34 | 2.01±0.36 |
| 7 | 3.12±0.14 | 2.67±0.29 | 3.54±0.14 | 2.48±0.33 |
| 28 | 2.07±0.06 | 1.68±0.09 | 1.99±0.13 | 1.12±0.11 |

**Results**

*Additional file 1 Figures and Figure Legends*

**Additional file 1 Figure 1:** The overview of experimental design and timepoints each technique was carried out. (A) Four parts of experimental design in the present study. (B) Images for histological analysis were obtained around the hematoma area marked with 1, 2 and 3 in the basal ganglia

**
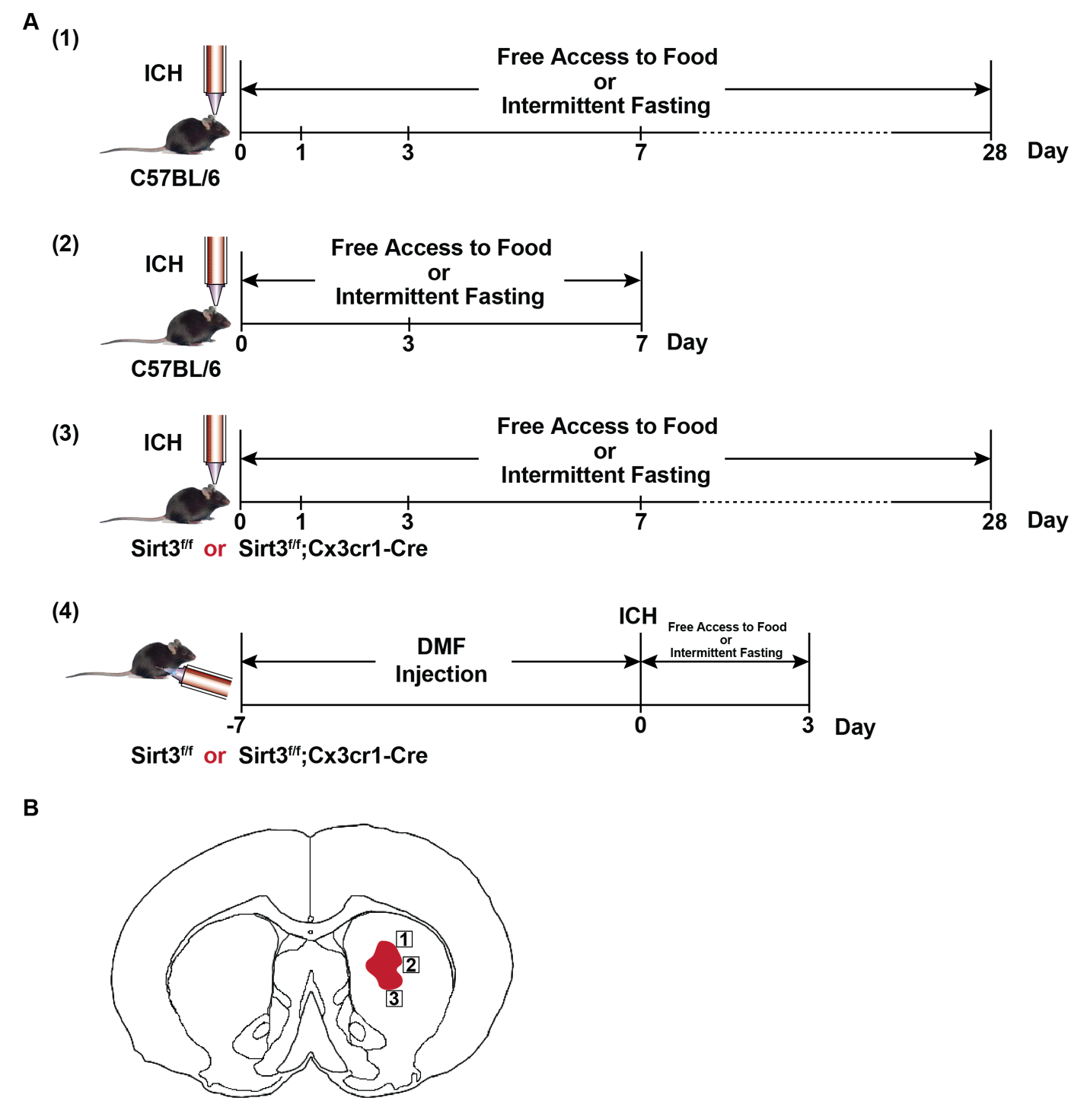
**

**Additional file 1 Figure 2:** Immunofluorescence staining and confocal microscopy for cleaved caspase-3 and NeuN. (A) Double fluorescent staining of cleaved caspase-3 (CCasp3, Green) and NeuN (Red) in the ipsilateral basal ganglia on days 1, 3 and 7, Scale bar=20μm. (B) Evaluation of CCasp3^+^NeuN^+^ cell numbers (n=6). Statistical analysis was performed using one-way ANOVA followed by post hoc Tukey’s test for multiple comparisons. Values are mean ± SD,  ^*^*P<*0.05 compared with ICH group.

**
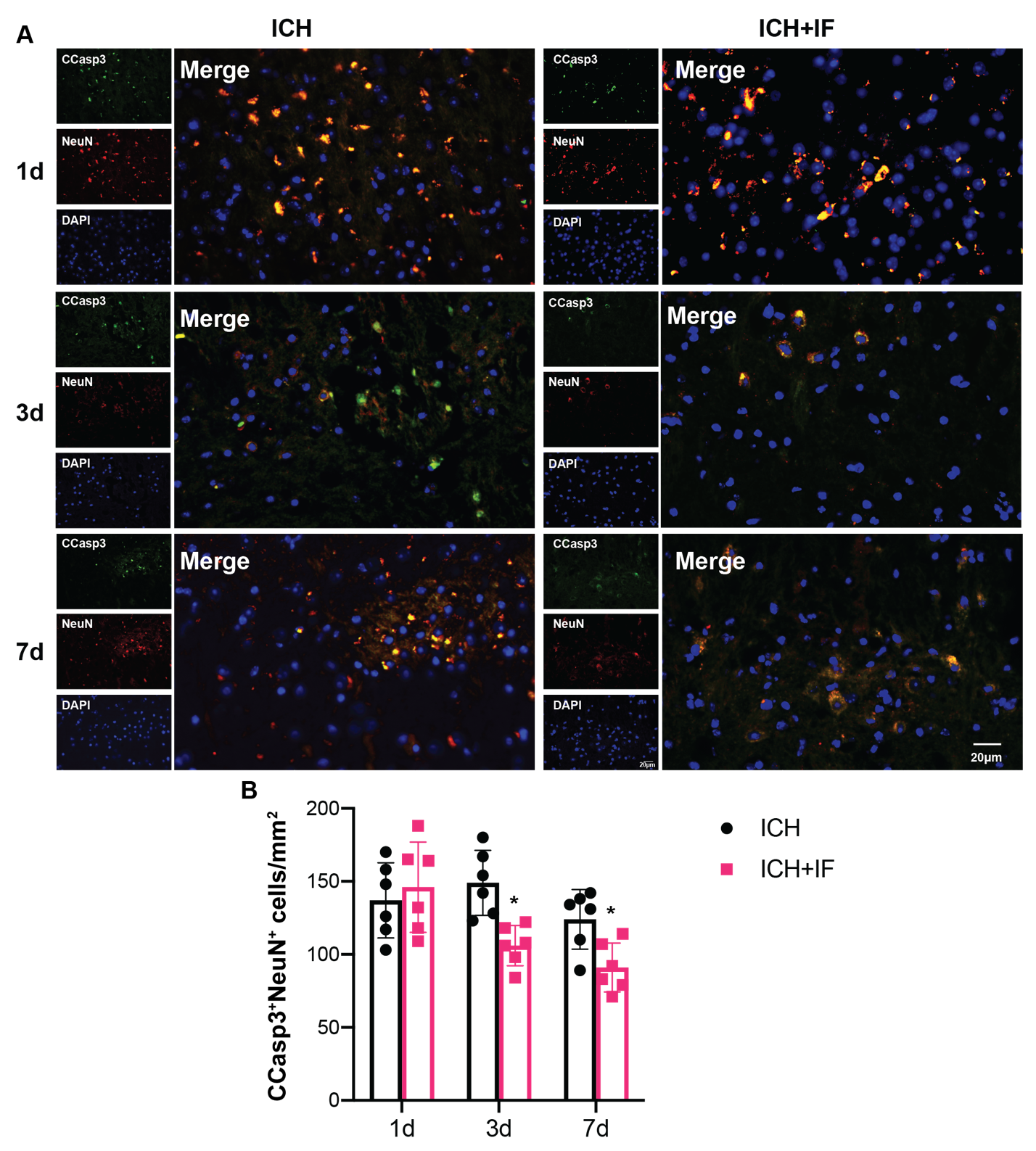
**

**Additional file 1 Figure 3:** Western blot analysis of Iba-1 protein expression in basal ganglia. (A) Western blot analysis of Iba-1 protein levels in the contralateral basal ganglia at day 1 after ICH and ipsilateral basal ganglia at day 1, 3, 7 and 28 in ICH and ICH+IF groups. (B) Quantification of relative gray value to control group (n=3). Statistical analysis was performed using one-way ANOVA followed by post hoc Tukey’s test for multiple comparisons. Values are mean ± SD, *^*^P*<0.05 compared with ICH group.

**
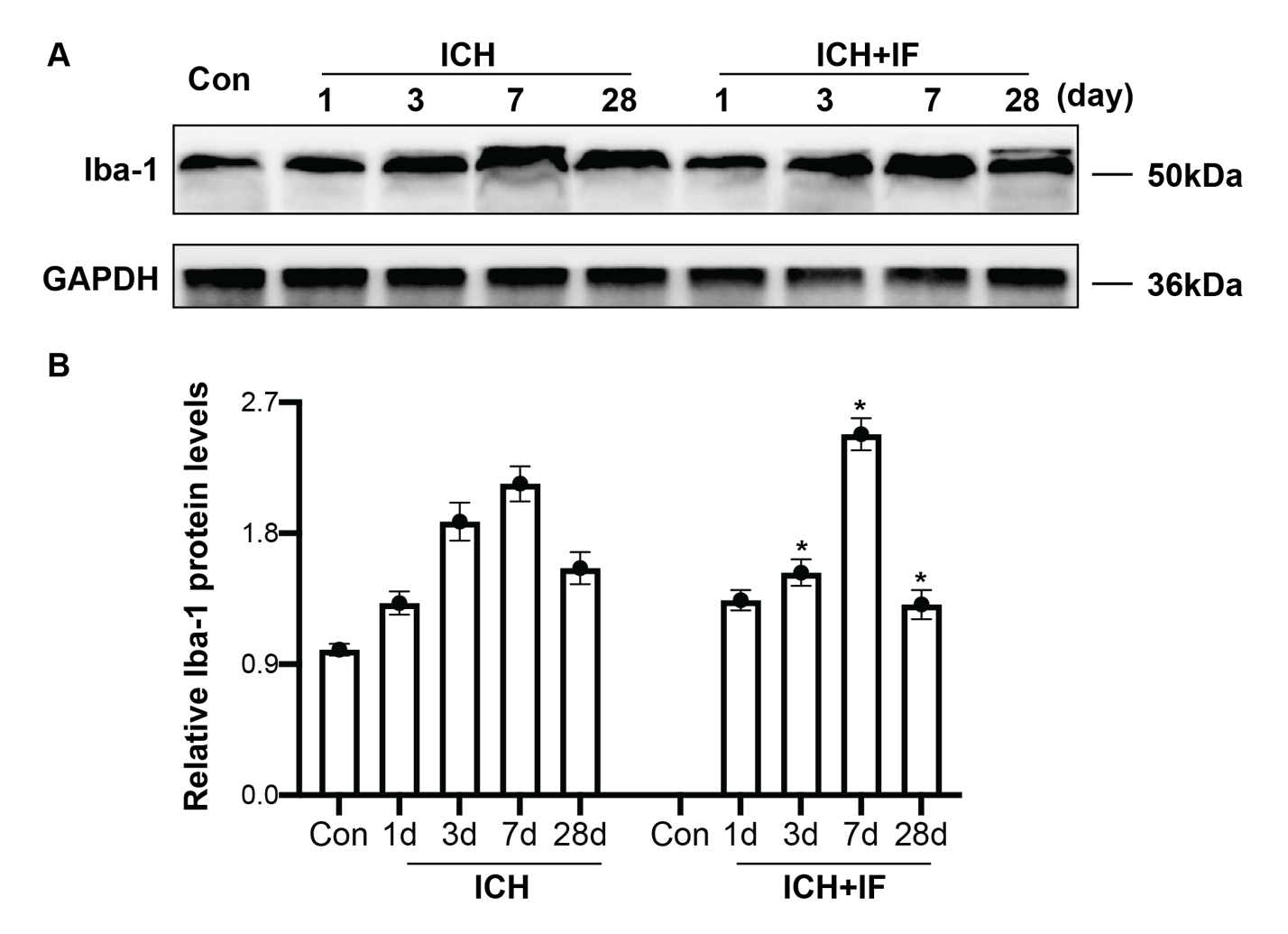
**

**Additional file 1 Figure 4:** Sirt3 expression in microglia of the ipsilateral basal ganglia after 7 day in ICH+IF group. (A) Double fluorescent staining of Sirt3 (Red) and Iba-1 (Green) in the ipsilateral basal ganglia, Scale bar=20μm. (B) Evaluation of fluorescence intensity of Sirt3 in the Sirt3^+^Iba-1^+^ cells (n=6). Statistical analysis was performed using Student *t* test. Values are mean ± SD, ^*^*P<*0.05 compared with ICH group.


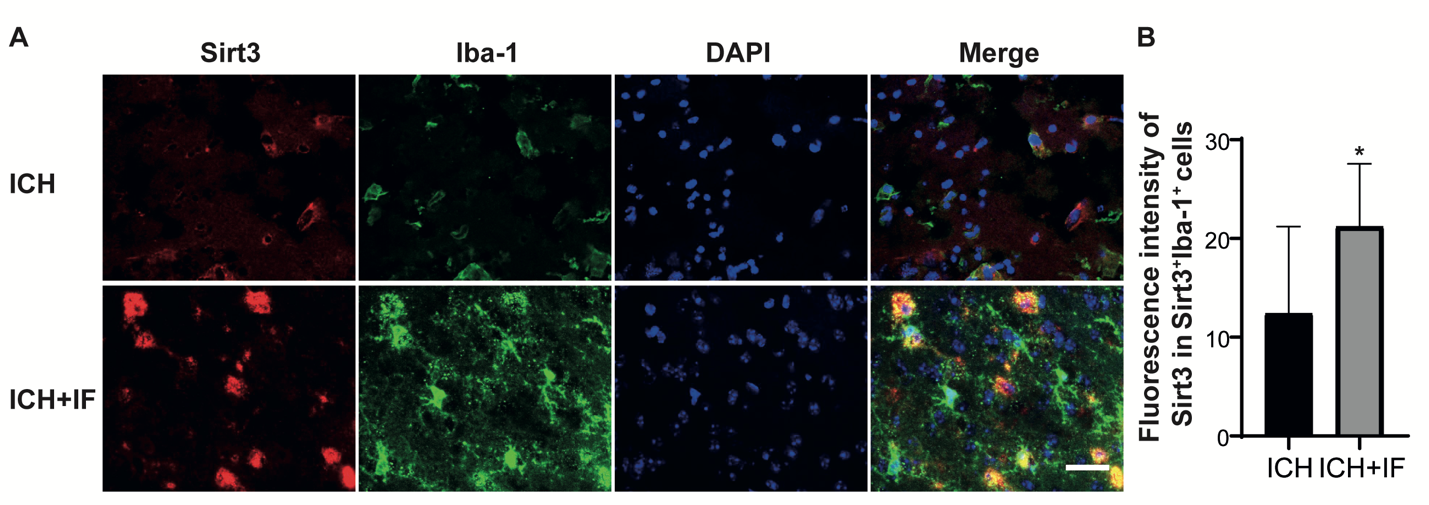


**Additional file 1 Figure 5:** Immunofluorescence staining and confocal microscopy for cleaved caspase-3 and NeuN. (A) Double fluorescent staining of CCasp3 (Green) and NeuN (Red) in the ipsilateral basal ganglia on days 1, 3 and 7 in Sirt3^f/f^ and Sirt3^f/f^;Cx3cr1-Cre mice after ICH+IF, Scale bar=20μm. (B) Evaluation of CCasp3^+^NeuN^+^ cell numbers (n=6). Statistical analysis was performed using one-way ANOVA followed by post hoc Tukey’s test for multiple comparisons. Values are mean ± SD,  ^#^*P<*0.05 compared with Sirt3^f/f^ group.


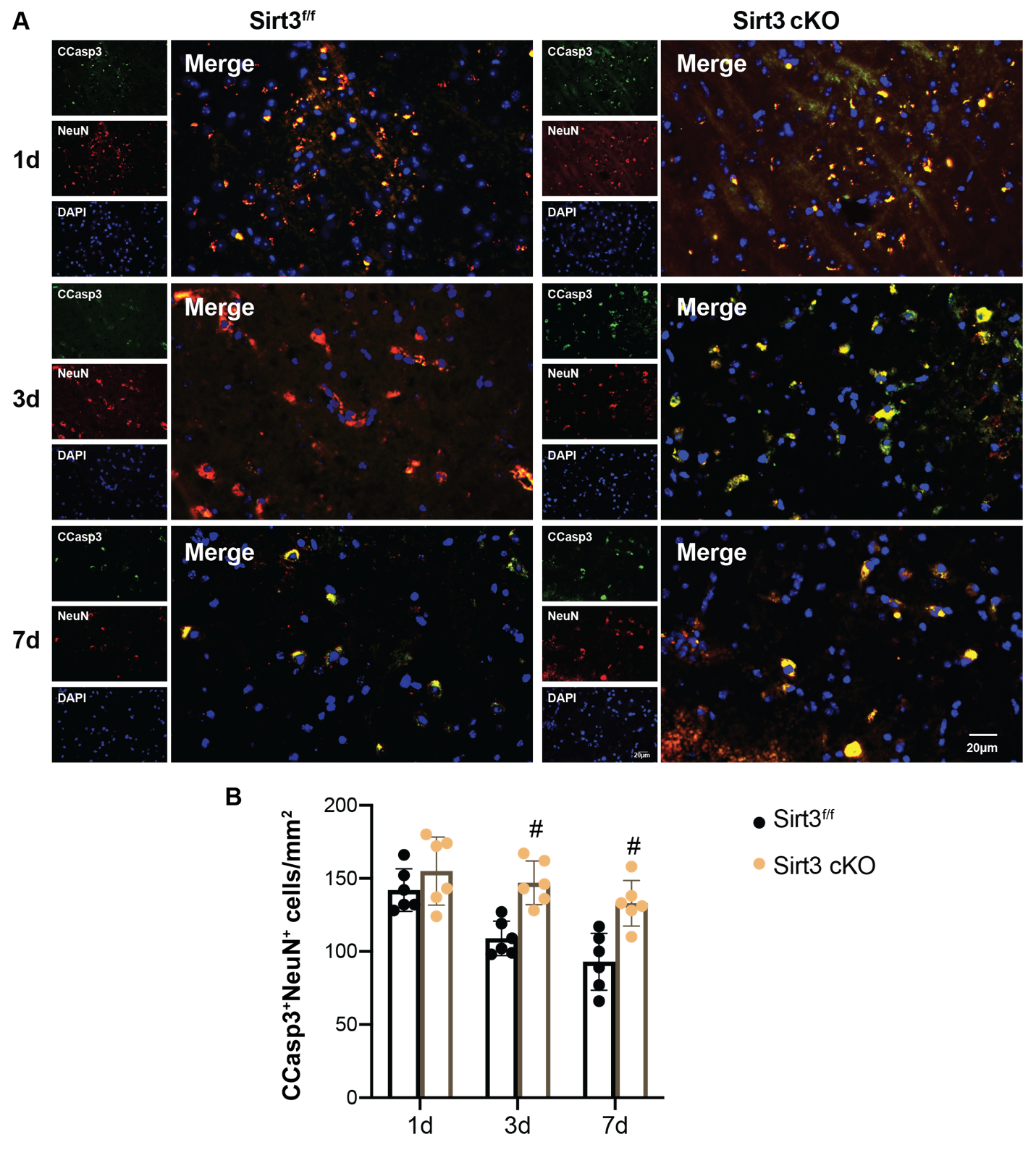


**Additional file 1 Figure 6:** Western blot of Nrf2/HO-1 expression in microglia of the ipsilateral basal ganglia after ICH.


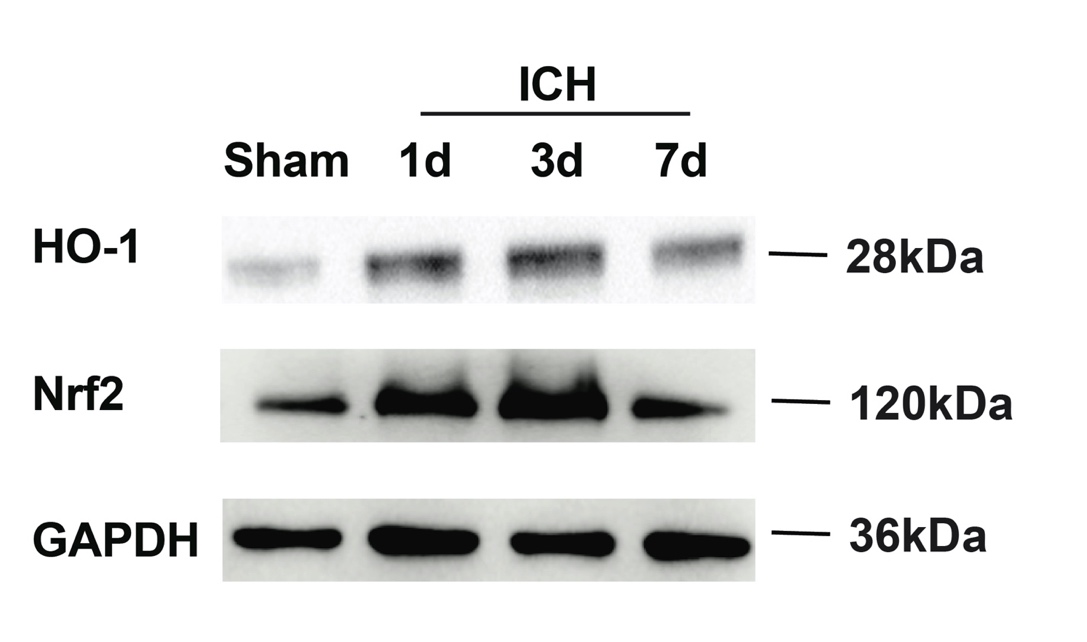


**Additional file 1 Figure 7:** Protective effects of DMF in Sirt3^f/f^;Cx3cr1-Cre mice at day 3 after ICH+IF. Cylinder test (A) and corner test (B) tests were performed pre-ICH and at day 3 after ICH+IF in Sirt3^f/f^;Cx3cr1-Cre mice (n=6) pre-treated with vehicle or DMF injection. (C) Representative HE staining images of the largest clot at day 3 after ICH+IF pre-treated with vehicle or DMF, Scale bar=1mm. Quantification of hematoma size (D) in Vehicel and DMF groups (n=6). Statistical analysis was performed using one-way ANOVA followed by post hoc Tukey’s test for multiple comparisons (A, B) and Student *t* test between two groups (D). Values are mean ± SD, ^+^*P*<0.05 compared with Vehicle group.


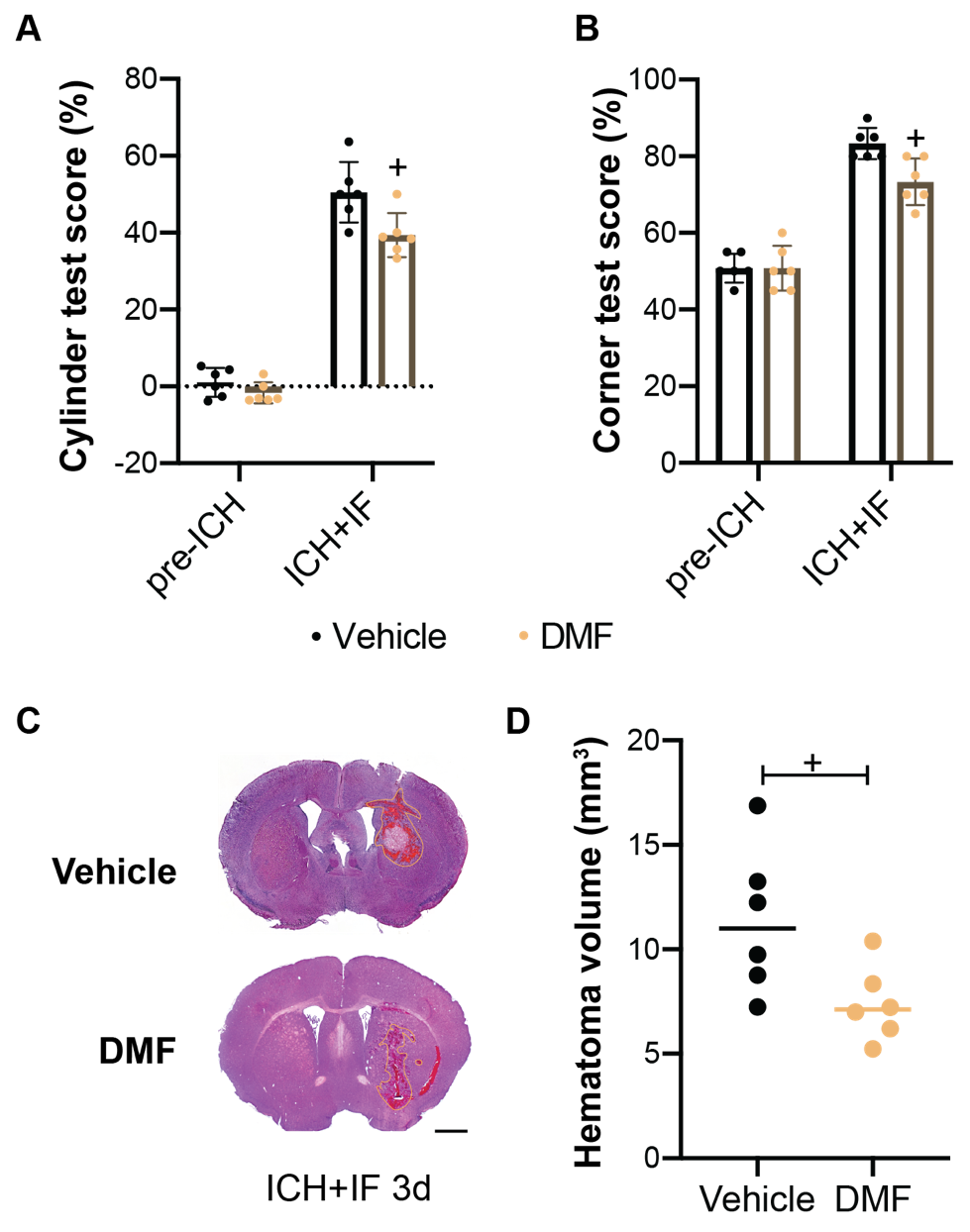


*Additional file 1 Tables*

**Additional file 1 Table 9:** The study design of different groups and numbers of mice in each group.

| Mice  Treatment | C57BL/6 | Sirt3^f/f^ | Sirt3 cKO |
| --- | --- | --- | --- |
| Sham | 6 |  |  |
| ICH | 42 | 9 | 9 |
| ICH+IF | 41 | 40 | 41 |
| ICH+IF+Vehicle |  | 6 | 13 |
| ICH+IF+DMF |  | 7 | 12 |
| Total number | 89 | 62 | 75 |

**Additional file 1 Table 10:** Primer sequences used for RT-PCR reaction.

| Gene | Forward primer (5′‐3′) | Reverse primer (5′‐3′) |
| --- | --- | --- |
| IL-1β | TACAGGCTCGTGCAGGACTCAG | GGTGGTGCGGCTGGATTGC |
| TNF-α | GCACTGAGAGCATGATCCGAGAC | CGACCAGGAGGAAGGAGAAGAGG |
| IL-10 | GGTGATGCCACAGGCTGAGAAC | GCTCCACCGCCTTGCTCTTG |
| TGF-β1 | AACCTACCCGACTGGTATC | CACAGCCGGACCTTTAAC |
| GAPDH | GGAAGCTGTGGCGTGATGGC | TTCTCCAGGCGGCAGGTCAG |
